# Forensic DNA phenotyping: a review on SNP panels, genotyping techniques, and prediction models

**DOI:** 10.1093/fsr/owae013

**Published:** 2024-03-11

**Authors:** Nuria Terrado-Ortuño, Patrick May

**Affiliations:** Luxembourg Centre for Systems Biomedicine, Genome Analysis, Bioinformatics Core, Esch-sur-Alzette, Luxembourg; Luxembourg Centre for Systems Biomedicine, Genome Analysis, Bioinformatics Core, Esch-sur-Alzette, Luxembourg

**Keywords:** forensic sciences, forensic DNA phenotyping, SNP panels, prediction models, forensics

## Abstract

In the past few years, forensic DNA phenotyping has attracted a strong interest in the forensic research. Among the increasing publications, many have focused on testing the available panels to infer biogeographical ancestry on less represented populations and understanding the genetic mechanisms underlying externally visible characteristics. However, there are currently no publications that gather all the existing panels limited to forensic DNA phenotyping and discuss the main technical limitations of the technique. In this review, we performed a bibliographic search in Scopus database of phenotyping-related literature, which resulted in a total of 48, 43, and 15 panels for biogeographical ancestry, externally visible characteristics, and both traits inference, respectively. Here we provide a list of commercial and non-commercial panels and the limitations regarding the lack of harmonization in terms of terminology (i.e., categorization and measurement of traits) and reporting, the lack of genetic knowledge and environment influence to select markers and develop panels, and the debate surrounding the selection of genotyping technologies and prediction models and algorithms. In conclusion, this review aims to be an updated guide and to present an overview of the current related literature.

## Introduction

In the forensic field, the use of human DNA has been mostly centred around individual identification using short tandem repeats (STR) [1–4]. This is achieved by “traditional matching”, also called forensic DNA identification, which is based on the comparison of an unknown DNA profile, obtained from a biological sample found in the crime scene, with a known DNA profile [5–8]. However, in some cases there are no matches, or no known profiles from a person of interest to compare it with [6, 9]. Thus, if other options are not feasible, such as using eyewitness statements, dragnets, or familial searching, these cases remain unsolved [7–12].

To overcome this, a new intelligence method emerged in the early 2000s, following the increase of genome-wide association studies (GWAS) that link common genomic variations, in particular single nucleotide polymorphisms (SNPs), with diseases and other phenotypic traits [9, 12–17]. SNPs are base substitutions, insertions, or deletions, that are normally bi-allelic with low mutation rates and high heritability [18–21]. Moreover, the small size of their PCR amplicons makes them useful to analyse typically forensic degraded and low amount DNA samples [1, 11, 13, 19, 21–23]. These findings have a big forensic potential since the prediction of externally visible characteristics (EVC) and bio-geographical ancestry (BGA), together with sex and age estimation, can provide a somehow physical description of a sample’s donor [7, 8, 13, 17, 18, 24]. Hence, the so-called forensic DNA phenotyping (FDP) (or molecular photo-fitting) aims to act as a “biological witness” [2, 25], providing new leads and reducing the pool of potential suspects [9, 11, 17]. FDP is also useful in missing persons’ investigations and for the identification of human remains [16, 22, 26–32]. Even though it has already been applied in some forensic cases [33–35], it raises several ethical, legal, and social issues about the limits of its application, dividing the forensic community [7, 8, 10, 11, 36, 37].

As mentioned before, STR profiling is a well-established and regulated technique owing to the great efforts from scientists and law enforcement to establish validated protocols in all forensic laboratories and to create police databases that contain profiles from criminals and missing persons [1, 4, 8] (more information available on the STRBase website [38]). In contrast, due to the relatively new appearance of FDP, there is no standardization of methodologies [11, 39, 40]. For instance, several SNP typing techniques have been adapted to analyse a growing number of SNPs in a single run and to input forensic-type samples [40, 41]. Although TaqMan® polymerase chain reactions (PCR) and single base extension (SBE) coupled with capillary electrophoresis (CE) (in particular, SNaPshot™ minisequencing) is extensively used, many efforts are now focused on implementing next generation sequencing (NGS) protocols [8, 11, 13, 17, 41].

In the last years, the available literature regarding FDP has grown exponentially: several reviews on new forensic developments started to include a small presentation of FDP [1–4, 17, 21, 22, 25, 27, 42–46]. Nonetheless, the number of articles exclusively dedicated to FDP is limited and not many evaluate in depth BGA [7, 47–49] or EVC (e.g. pigmentation traits [7, 24, 36, 50–52] and other characteristics such as weight, height, or facial morphology [8–12, 14, 53, 54]). Phillips' review [49] is one of the few to include a comparison of BGA-informative markers and panels worth of consideration for forensic application, while reviews of Mehta et al.'s [13] and Schneider’s [7] reviews describe the most informative panels for both BGA and EVC inference. The latest reviews on the current state of EVC prediction have been published by Tozzo et al. [8], Pośpiech et al. [12], and Dabas et al. [54]. All these include an extensive summary on newly found genetic markers and most common panels to predict continental, sub-continental and admixed ancestries, pigmentation traits (eye, hair and skin colour, hair greying), hair morphology (shape and thickness), eyebrow morphology (colour, thickness, monobrow), height, weight (BMI), facial morphology, presence of freckles, male-pattern baldness, and myopia. They also give a few comments on different prediction algorithms, genotyping technologies, and some limitations in FDP.

Despite this, there are currently no publications that gather all the existing research limited to FDP. Thus, this review will include only those articles that have specifically developed and/or applied panels with the aim to use it as an intelligence tool to reduce the set of suspects or to identify human remains, excluding those regarding the discovery of markers in a non-forensic/clinical setting. This will allow us to have a general view of all the commercial, commonly used, and other customized sets of markers and to provide an evaluation of the FDP field. In particular, the focus will be on the lack of harmonization concerning classification algorithms and methodologies, and limitations in terms of reference datasets, informative SNPs, environmental influences, and lack of a common lexicon, among others. To do so, we will take as reference the following reviews [7, 8, 12, 13, 52, 54] and present a more detailed summary that will include the panels’ markers (type and number), genotyping technology, statistical methods, traits, and related literature. Hence, the aim of this scoping review is to present an overview of the current FDP-related literature, so it serves as an updated guide of the global aspects of FDP that can redirect to further specific reading.

## Materials and methods

This review followed the Preferred Reported Items for Systematic Reviews and Meta-Analyses extension for Scoping Reviews (PRISMA-ScR) guidelines [55].

Any published paper, written in English, between 2000 and 2022, and whose focal point was FDP (in particular, EVCs and BGA) were eligible for inclusion. It is important to notice that only those papers that researched genetic human variation for FDP applications using SNPs were considered, whilst those that referred to the ethical, legal, and social implications, were not because they are beyond the scope of this review.

Four separate searches were performed on Scopus database (last search in January 2023). First, a generalized search was carried out as follows: “forensic DNA phenotyping” OR “forensic DNA intelligence” OR “molecular photo-fitting”. To obtain a more specialized search on the topic, the other four searches were conducted with the following combination of keywords: (1) “external visible characteristics” OR “physical appearance” OR “physical trait” OR “physical characteristic” AND “forensic”; (2) “biogeographical ancestry” AND “forensic”; and (3) “SNP typing” OR “prediction model” AND “forensic”. A total of 1 016 records were obtained from Scopus (FDP = 376, EVC = 241, BGA = 97, and methods = 302). After removal of duplicates (*n* = 77), a manual selection of documents was first performed based on title and abstract and after on a full text evaluation. The following criteria were used to select the articles: if they inferred BGA or/and EVC, if they specified their aim was for forensic phenotyping and not identification purposes, if the analysis was performed with human DNA samples/data, if the main marker type was SNPs and if the manuscript was available and not retracted. It concluded with the inclusion of 201 articles. Finally, 101 records were identified from the chosen papers’ references. For each article, author(s), title, year of publication, publication journal, and details on their studied FDP trait can be found in the Supplemental material.

## Discussion

### BGA

BGA describes the most likely continental and/or sub-continental regions of origin of an individual’s ancestors. Despite it being based on the genomic differences and similarities among populations [1, 56], it should not be confused with the notion of ethnicity, nationality, or religious affiliations since it does not represent the place of birth or where one lives [7, 57, 58].

Although STRs were the first markers proposed to infer someone’s origins [59, 60], SNPs show greater inter-population differences and a positive association with ancestral populations [3, 18]. Current research is focused on combining different types of markers, such as insertions/deletions (InDels) [61] and microhaplotypes (MH) [62] with SNPs, especially for the analysis of DNA mixtures and admixed individuals, respectively [48]. However, in this review, only those panels including SNPs will be considered.

There are three types of SNPs considered as ancestry-informative markers (AIMs or AISNPs): Y-chromosome SNPs (Y-SNPs), mitochondrial SNPs (mt-SNPs), and autosomal SNPs (aSNPs). The first two define paternal and maternal haplogroups and they have been historically used for evolution studies because of their low recombination rates, their non-random geographical distribution, and their well-known global frequency distribution [63, 64]. Interestingly, Y-SNPs show a better genetic differentiation with geographical distance than mt-SNPs or aSNPs due to patrilocality [5]. The main issue when inferring ancestry using non-autosomal markers is that although being highly accurate when recent ancestors were from the same region [65], they only represent half of the lineage [48] and they can lead to misinterpretation of complex origins [49]. Therefore, aSNPs are proposed in combination with parental SNPs to infer admixed ancestries [7, 21, 35, 48, 66, 67].

#### BGA-related literature

In 2001, Jobling [5] published the first review that considered Y-SNP haplogroup inferring as an exclusion tool to target an initial suspect. Many reviews that discuss the aspects of BGA inference such as the development of panels and selecting classification algorithms are available [47, 48, 67–70]. However, only few discuss the different panels for FDP application [7, 13, 49], and they are usually centred around the most used ones. In this review, a total of 48 sets of markers that have been developed and/or applied in forensic genetics can be found in the Tables 1 and 2.

**Table 1:** Non-commercial bio-geographical ancestry (BGA) panels proposed in the literature, including their reference article, number and ancestry informative SNPs (AISNPs) type, first used genotyping technology and prediction model, inferred populations, and related articles.

	**AISNPs**	**Genotyping technology**	**Statistical model**	**Inferred BGA**	**Related articles**
**Y-AISNPs panel**					
Major Y-chromosome haplogroup typing kit [216, 217]	29 Y-SNPs	SNaPshot™ + CE	MDS	31 major global Y-haplogroups	[218]
[219]	30 Y-SNPs	SNaPshot™ + CE	MDS	32 major EUR Y-haplogroups	NA
[220]	37 Y-SNPs	SNaPshot™ + CE	CRT	Major EUR Y-haplogroups	NA
[221]	13 Y-SNPs	SNaPshot™ + CE	GDA CRT NB (Snipper)	Major ASN Y-haplogroups	NA
[222]	12 Y-SNPs	SNaPshot™ + CE	CRT	Venezuelan Y-haplogroups	NA
[223]	28 Y-SNPs	SNaPshot™ + CE	CRT	Macedonian Y-haplogroups	NA
[224]	28 Y-SNPs	SNaPshot™ + CE	CRT	Major global Y-haplogroups	NA
[159]	7 Y-SNPs	SNaPshot™ + CE	CRT	EUR, EAS, AFR Y-haplogroups	NA
[225]	859 Y-SNPs	NGS	CRT	640 Y-haplogroups	NA
[182]	9 Y-SNPs	PCR-REBA + Sequencing	CRT	Major global Y-haplogroups	NA
**mt-AISNPs panel**					
[226]	11 mt-SNPs 1 mt-InDel	SNaPshot™ + CE	CRT	15 mt-haplogroups	NA
[227]	36 mt-SNPs	SNaPshot™ + CE	CRT	43 mt-haplogroups (AFR, west and east EURAS, NAM)	NA
[228]	26 mt-SNPs	SNaPshot™ + CE	CRT	20 OCE and 10 AFR, EUR and ASN mt-haplogroups	NA
[229]	62 mt-SNPs	SNaPshot™ + CE	CRT	70 global mt-haplogroups (AFR, NAM, WEAS, EAS, AUS, OCE)	NA
[230]	52 mt-SNPs	SNaPshot™ + CE	CRT	Major global mt-haplogroups	[230]
**aAISNPs panel**					
[231]	6 aSNPs	SNaPshot™ + CE	NJ	Major AUS sub-populations	NA
SNPforID 34-plex [72, 156, 232]	34 aSNPs	SNaPshot™ + CE	NB (Snipper and STRUCTURE)	3 populations (AFR, EUR, EAS)	[78, 187, 188, 232–237]
[66]	176 aSNPs	SNPstream + CE	NB (STRUCTURE) ML (unspecified)	4 populations (EUR, WAF, NAM, EAS)	NA
[169, 238]	16 aSNPs	SNaPshot™ + CE	NB (Snipper)	6 AUS sub-populations	NA
[151]	47 aSNPs	GeneChip® array TaqMan® SNP genotyping	NB (STRUCTURE)	4 populations (AFR, EURAS, EAS, NAM)	NA
Seldin set [136]	128 aSNPs	TaqMan® SNP genotyping	NB (STRUCTURE)	4 populations (AFR, EUR, EAS, NAM)	NA
[170]	16 aSNPs	SNaPshot™ + CE	MLR	7 populations (WAF, NAF, Turkey, NES, Balkan states, NEU, Japan)	NA
Eurasiaplex [74]	23 aSNPs	SNaPshot™ + CE	NB (Snipper and STRUCTURE)	2 sub-populations (EUR and EAS, MES, and SAS)	[187, 188, 232]
EUROFORGEN Global AIM-SNP [78]	128 aSNPs	Sequenom® MassARRAY® Sanger sequencing	MDS NB (Snipper and STRUCTURE)	5 populations (AFR, EUR, EAS, NAM, OCE)	[183, 239]
Kidd Lab [77]	55 aSNPs	TaqMan® SNP genotyping	MDS NB (STRUCTURE)	7 to 8 populations (sub-Saharan AFR, admixed and NEAF, SWAS, EUR, Siberian, SAS, EAS, SEAS, PAC, NAM)	[240]
[163]	14 aSNPs	SNaPshot™ + CE	NB (Snipper) SVM	3 populations (EUR, AFR, EAS)	NA
EurEAs_Gplex [241]	14 aSNPs	SNaPshot™ + CE	MDS NB (Snipper and STRUCTURE)	3 populations (EUR, AFR and EAS)	NA
Global AIMs Nano [157]	31 aSNPs	SNaPshot™ + CE	NB (Snipper and STRUCTURE)	5 populations (AFR, EUR, EAS, OCE, NAM)	NA
[242]	32 aSNPs	TaqMan® SNP genotyping	NB (STRUCTURE)	MED and SWAS	NA
[243]	74 aSNPs	TaqMan® SNP genotyping Sequenom® MassARRAY®	NB (STRUCTURE)	10 populations (sub-Saharan AFR and NAF, EUR, SWAS, NAS, SAS, EAS, SEAS, OCE, NAM)	[186]
[184]	130 aSNPs	Sequenom® MassARRAY®	MLR	EUR and 5 ASN sub-populations	NA
[68]	142 aSNPs	Not used	NB (Snipper and STRUCTURE) MLR GDA	4 populations (AFR, EUR, EAS, NAM)	[199]
[67]	93 aSNPs	Not used	NN	7 populations (AFR, EUR, CSAS, MEA, EAS, NAM, OCE)	NA
Japaneseplex [244]	60 aSNPs	SNaPshot™ + CE	NB (Snipper)	EAS sub-populations	NA
SWA AISNP panel [58]	86 aSNPs	TaqMan® SNP genotyping	NB (STRUCTURE)	SWAS and MED sub-populations	[240]
EUROFORGEN NAME [79]	111 aSNPs	Sequenom® MassARRAY®	NB (Snipper and STRUCTURE)	NAF and MES	[185]
[167]	48 aSNPs	Not used	NB (ADMIXTURE) MLR	Chinese sub-populations (Uygur, Han, Mongolian)	NA
Population Informative Multiplex for the Americas (PIMA) [76]	26 aSNPs	SNaPshot™ + CE	PCA, NB (Snipper)	NAM sub-populations	[245]
**Multiple AISNPs panel**					
[65]	7 Y-SNPs, 12 mt-SNPs, 6 aSNPs	SNaPshot™ + CE HRM	NB (STRUCTURE)	2 populations (ASN and EUR)	NA
[246]	31 aSNPs, 21 InDels	SNaPshot™ + CE	NB (Snipper and STRUCTURE)	5 populations (AFR, EAS, MES, EUR, CSAS)	NA
Pacifiplex [75]	27 aSNPs, 2 X-SNPs	SNaPshot™ + CE	NB (Snipper and STRUCTURE)	OCE sub-populations	[187, 188, 236]
MAPlex [247, 248]	144 aSNPs, 20 MH	NGS	NB (Snipper and STRUCTURE)	3 populations (EAS, SAS, NOCE)	[249]

EUR: European (NEU: North); AFR: African (WAF: West, NAF: North, NEAF: North-east); ASN: Asian (EAS: East, WEAS: West–east, SAS: South, SWAS: South-west, SEAS: South-east, central-south, NAS: North); EURAS: Eurasian; NAM: Native American; AUS: Australian; OCE: Oceanian (NOCE: Near); PAC: Pacific; NES: Near East; MES: Middle East; MED: Mediterranean; NGS: next generation sequencing; MDS: multidimensional scaling; CRT: classification and regression tree; GDA: genetic distance algorithm; NJ: neighbour joining tree; NB: Naive Bayes; ML: Machine learning; MLR: Multinomial linear regression; SVM: support vector machine; CSAS: central-south Asian; MES: middle eastern; NA: not applicable/available.

**Table 2:** Commercial bio-geographical ancestry (BGA) panels proposed in the literature, including their reference article, number and AISNPs type, first used genotyping technology and prediction model, inferred populations, and related articles.

	**SNPs**	**Genotyping technology**	**Statistical model**	**Inferred BGA**	**Related articles**
Signet™ Y-SNP kit (Marligen Bioscience Inc.)	42 Y-SNPs Amelogenin	Multiplex PCR + flow cytometry	CRT	6 major global Y-haplogroups	[173]
Precision ID Ancestry Panel (ThermoFisher Scientific)	165 aSNPs	NGS	HID-SNP Genotyper PlugIn (undisclosed)	7 populations (EUR, AFR, NAM, EAS, OCE, SAS, SWAS)	[35, 57, 160, 168, 191, 195, 214, 250–260]
DNAWitness™ (DNAPrint Genomics)	178 aSNPs	SNPstream®	NA	4 populations (sub-Saharan AFR, NAM, EAS, EUR)	NA
DNAWitness-Y™ (DNAPrint Genomics)	NA	SNPstream®	NA	Y-haplogroups	NA
DNAWitness-Mito™ (DNAPrint Genomics)	NA	SNPstream®	NA	mt-haplogroups	NA
EUROWitness™ (DNAPrint Genomics)	NA	SNPstream®	NA	4 EUR sub-populations (NWEU, SWEU, MES and SAS)	NA

European (NWEU: North-west, SWEU: South-west); AFR: African; ASN: Asian (EAS: East, SAS: South, SWAS: South-west); NAM: Native American; OCE: Oceanian; MES: Middle East, NGS: next generation sequencing; CRT: classification and regression tree; HID: human identification; NA: not applicable/available.

The first commercially available tools for forensic inference of BGA were launched in 2003 by DNAPrint Genomics: DNAWitness-Y™ and DNAWitness-Mito™ for parental lineages, and DNAWitness™ to infer sub-Saharan African, Native American, East Asian, and European ancestries (the latest also can be sub-divided into North-western European, South-western European, Middle Eastern, and South Asian using the EUROWitness™ panel). These panels had already been applied to solve real forensic cases, such as the Louisiana rapist or the Night Stalker [33]. After they were discontinued, a well-known NGS-based commercial solution was presented by ThermoFisher Scientific: The Precision ID Ancestry Panel (before known as HID-Ion AmpliSeq™ Ancestry Panel) [71], which includes 165 aAISNPs, allowing the differentiation of African, European, American, East Asian, South Asian, Southwest Asian, and Oceanian populations.

Concerning the non-commercial panels, one of the first applied panels in forensic research was proposed by the SNPforID Consortium. The SNPforID 34-plex panel [72] allows differentiation among sub-Saharan Africans, Europeans and East Asians and is suitable to use with SNaPshot™ technology. This panel is included in the online webtool Snipper App, developed by the University of Santiago de Compostela (USC) [73] and it allows to use the panel to infer three to five populations and to choose a classifier among naïve Bayes (NB; applying or not Hardy–Weinberg equilibrium (HWE)), multinomial logistic regression (MLR), or genetic distance algorithm (GDA) (according to allele and genotype frequency).

The following years, three population-specific panels were developed to be used in combination with the 34-plex: a 23-plex called Eurasiaplex [74], which enhances differentiation between Europeans and South Asians; the Pacifiplex [75], a panel of 29 AIMs for differentiating Oceanian populations; and the 26-plex Population Informative Multiplex for the Americas (PIMA) dedicated to Indigenous American populations [76]. Other most used sets in this field are the Kidd’s lab panel, containing 55 SNPs that can distinguish seven to eight continental regions [77], and the EUROFORGEN Global AIM-SNP set which is composed of 128 autosomal SNPs to differentiate the main five global groups (Africa, Europe, East Asia, Native America, and Oceania) [78]. This last panel was reduced to a 31-plex, the Global AIMs Nano, and can be combined with the EUROFORGEN NAME [79], which uses 111 aSNPs to enhance differentiation of Middle Eastern and North Africans.

### EVC

EVC are described as physical traits that are apparent at view (i.e. pigmentation, height, weight, and facial morphology). Genetically, they are considered complex traits due to their multigenic and multifactional nature [11, 14, 80], since they are influenced by environmental factors [11, 17], such as climate, altitude, and nutrition [1, 6, 14, 36]. The markers used to infer EVC are commonly referred to as phenotypic-informative SNPs (PISNPs) and can be both present in the coding and non-coding regions of the DNA [21].

The pigmentation variation depends on the amount, type, and distribution of melanin and eumelanin and it varies depending on the sex, age, ultra-violet ray (UVR) exposure and body site [24, 50, 80–82]. It is said that their genetics follow a semi-Mendelian inheritance [11, 24], and their heritability is between 60% and 90% [7, 12, 14]. For this very reason, they have been the focus of FDP—since their genetics is extensively studied in clinical research—and they were the first ones to be predicted. The eye colour is the most successfully predicted EVC and is highly variable in European ancestries [43, 83]. It is normally categorized in two or three groups: blue (non-brown) *vs* brown (non-blue), or blue *vs* intermediate (green/hazel) *vs* brown/black. Additionally, they are divided into light (blue, green) and dark categories (brown, black). Instead, hair colour is usually divided into blond, brown, red, and black groups. This trait is highly influenced by age, since individuals with red and blond hair in their childhood usually transition to blond and brown—respectively—, and hair whitens/greys when older [6, 9, 11, 24, 43]. In addition, the categorization of skin colour in humans is the most complex and the most varying among studies. Usually, they are divided according to the Fitzpatrick Scale [84] as very pale *vs* pale/light *vs* intermediate/olive/light-brown *vs* brown *vs* dark/black. For this reason, skin colour is the most complicated trait to predict among the pigmentation phenotypes [21, 43]. Another unique pigmentation feature is the presence of ephelides—or freckles—which is also affected by URV exposure and age [85, 86]. Their prediction can be based on a two- and four-categorical model: non-freckled *vs* freckled, or light-freckled *vs* mild-freckled *vs* severe-freckled *vs* non-freckled.

Two of the most interesting quantitative traits are height and weight. On one hand, stature is an easy-to-measure trait, leading to homogeneous, reliable, and accurate data [12, 14]. It is highly polygenic and greatly influenced by environmental factors (e.g. social class, income, education, family size, housing, urban locations, etc.) [9, 14]. Although it has been immensely studied in clinical research, few studies are directed to its incorporation to FDP. On the other hand, an individual’s weight is usually measured using the BMI, and it is said to have a heritability around 60%–70%, despite knowing that epigenetic factors have more influence that genetic ones [87].

The most ambitious and challenging phenotype to incorporate is facial morphology. Even though environment has a small effect, and the genetic component is strong, it is highly polygenic, and the knowledge of their underlying genetic mechanisms is scarce [8, 9, 11, 12, 88]. The FaceBase Consortium [89] and the International Visible Trait Genetics (VisiGen) Consortium [90] have discovered many markers associated with the actual human morphology and researchers tend to first focus on single facial features to later apply it to whole-face predictions [11, 16, 88, 91]. Some of the individual traits that have been investigated are related to eye morphology, such as eyelid fold, epicanthal index, and palpebral fissure distance and inclination.

Another trait that could be incorporated in FDP is hair morphology, including hair shape, which can be grouped into three categories: straight, wavy, and curly; hair thickness from the scalp hair; eyebrows (e.g. monobrows) and beard; and hair loss, in particular male-patterned baldness (MPB). Moreover, an interesting phenotype that has only been suggested once [92] is the relative hand skill (HSR) or handedness, which is based on the preference of using the right or left hand to perform complex tasks.

#### EVC-related literature

The prediction of physical characteristics is being extensively researched, in comparison with BGA. There are many reviews solely focused on the current knowledge on genetic mechanisms of pigmentation traits and facial morphology, as well as discovering new and more informative markers [93–106], and few include other traits that have potential to be included as part of FDP (freckles [96, 103, 107, 108], facial morphology [109–113], high myopia [114], handedness [92], hair greying [115, 116] or hair morphology [26, 116–118]). In terms of prediction panels, there are seven reviews that include detailed descriptions on traits and their associated SNPs [7, 8, 12, 13, 15, 52, 54]. This review includes a total of 43 sets of markers to infer EVC (Table 3).

**Table 3:** Commercial and non-commercial externally visible characteristics (EVC) panels proposed in the literature, including their reference article, number of phenotypic-informative SNPs (PISNPs), first used genotyping technology and prediction model, inferred traits, and related articles

	**SNPs**	**Genotyping technology**	**Statistical model**	**Inferred traits**	**Related articles**
**Pigmentation traits**					
RETINOME™ (DNAPrint Genomics)	NA	NA	NA	Eye colour	NA
IrisPlex [83, 261]	6 PISNPs	SNaPshot™ + CE	MLR	Eye colour	[35, 81, 82, 104, 139, 142–146, 179, 187, 188, 200, 208, 210, 212, 245, 262–274]
SHEP 1 [212]	13 PISNPs	SNaPshot™ + CE	NB (Snipper)	Eye colour	[145, 208, 237, 245, 269, 270]
[275]	19 PISNPs	TaqMan® SNP genotyping	CRT	Eye colour	[208]
[140]	23 PISNPs 2 InDels	TaqMan® SNP genotyping SNaPshot™ + CE	LR	Eye colour	NA
[276]	2 PISNPs	TaqMan® SNP genotyping	LR	Eye colour	NA
[277]	5 PISNPs	Sanger sequencing	MLR	Eye colour	NA
[198]	137 PISNPs	NGS	LR, CRT, RF, XGB, MARS, NN, SVM and NB	Eye colour	NA
EC11 [209]	11 PISNPs	Sequenom® MassARRAY®	LR, CRT	Eye colour	NA
HIrisPlex [120, 278]	23 PISNPs 1 InDel	SNaPshot™ + CE TaqMan® SNP genotyping	MLR	Eye colour Hair colour	[31, 139, 160, 195, 200, 279–284]
[201]	12 PISNPs	SNaPshot™ + CE	MLR, BLR, NB	Eye colour Hair colour	[273]
[285]	10 PISNPs 2 InDels	Solid-phase fluorescent minisequencing	GDA	Hair colour	NA
[286, 287]	5 PISNPs	SNaPshot™ + CE	GDA	Hair colour	NA
[288]	11 PISNPs Amelogenin	SNaPshot™ + CE	BN	Hair colour	NA
[154]	13 SNPs	Sequenom® MassARRAY® SNaPshot™ + CE	MLR, LASSO regression	Hair colour	[210]
SHEP 4 [233]	12 PISNPs	SNaPshot™ + CE	LR NB (Snipper, iterative NB)	Hair colour	[237]
[115]	12–14 PISNPs Amelogenin	NGS	NN	Hair greying	NA
HIrisPlex-S [121]	41 SNPs	SNaPshot™ + CE	MLR	Eye colour Hair colour Skin colour	[98, 139, 180, 189, 193, 194, 196, 200, 240, 258, 289–293]
[153]	13 PISNPs	SNaPshot™ + CE	MDR MLR	Eye colour Hair colour Skin colour	NA
[210]	12 PINSPs	SNaPshot™ + CE	LR, OR	Eye colour Hair colour Skin colour	NA
CAN-E, CAN-S, and CAN-H [207]	277 PISNPs	Not used	LR, MLR, RF, XGB, ANN, OR and SR	Eye colour Hair colour Skin colour	NA
[294]	12 PISNPs	TaqMan® SNP genotyping	MLR	Eye colour Hair colour Skin colour	NA
[295]	7 PISNPs	TaqMan® SNP genotyping	GDA	Eye colour Skin colour	[265, 296]
[296]	8 PISNPs	TaqMan® SNP genotyping	GDA	Eye colour Skin colour	[208, 297]
[298]	5 PISNPs	NGS	GDA	Eye colour Skin colour	NA
[202]	14 PISNPs	SNaPshot™ + CE	LR, RF and NN	Skin colour Tanning Freckles	NA
[86]	5 PISNPs	KASP Genotyping Chemistry TaqMan® SNP genotyping	MLR	Freckles	NA
[85]	12–14 PISNPs	NGS	LR	Freckles	[200]
SHEP 1 [253]	110 PISNPs	SNaPshot™ + CE	NB (Snipper)	Skin colour	[237]
**Other hair-related traits**					
[164]	6 PISNPs	SNaPshot™ + CE NGS	LR, CRT, and NN	Hair morphology	NA
[117]	32–33 PISNPs	NGS Sequenom® MassARRAY®	LR	Hair morphology	[200]
[299]	14 PISNPs	Microarray	MLR	Hair morphology	NA
[300]	4–21 PISNPs	NGS	LR	Hair morphology	NA
[155]	5–20 PISNPs	SNaPshot™ + CE NGS	LR	Male-pattern baldness	NA
[165]	25 PISNPs	SNaPshot™ + CE PCR-RFLP	LR	Male-pattern baldness	NA
**Facial traits**					
[88, 91]	24 PISNPs 68 AISNPs Amelogenin	SNPStream™	PLSR, BRIM	Facial morphology	NA
[301]	~ 90.000 PISNPs	Microarray	PCA, LR	Facial morphology	NA
[113]	1 PISNPs	NGS	LR	Eyelid	NA
[302]	4 PISNPs	Microarray	PCA	Facial morphology	NA
[303]	21 PISNPs	SNaPshot™ + CE	OR MLR	Ear morphology	NA
**Other traits**					
[87]	8 PISNPs 4 CpG sites	SNaPshot™ + CE Pyrosequencing	RF	BMI	NA
[161]	180 PISNPs	Microarray	LR	Height	NA
[162]	412–689 PISNPs	Microarray	LR	Height	NA

NA: not applicable/available; MLR: multinomial linear regression; NB: Naive Bayes; CRT: classification and regression tree; LR: likelihood ratio; RF: random forest; XGB: extreme gradient boosting; MARS: multi-variate adaptive regression splines; NN: neural networks; SVM: support vector machine; GDA: genetic distance algorithm; BN: Bayesian Networks; LASSO: least absolute shrinkage and selection operator; ANN: artificial neural networks; OR: ordinal regression; SR: step-wise regression; PLSR: partial least square regression; BRIM: bootstrapped response-based imputation modeling; PCA: principal component analysis.

The only commercial test purely focused on EVC inference was **RETINOME**™, also developed by DNAPrint™ Genomics who guaranteed a 97% of correct eye colour predictions [119]. Nonetheless, the most currently used free online tools to predict pigmentation traits are the **IrisPlex** system and its updated versions (**HIrisPlex** and **HIrisPlex-S**), created by the Erasmus University Medical Centre Rotterdam [83, 120–122]. IrisPlex uses six SNPs to predict blue, intermediate, and brown colours with an average accuracy of 0.94 area under the curve (AUC), 0.74 AUC and 0.95 AUC, respectively. The HIrisPlex system allows the inference of eye and hair colour by simultaneously targeting 23 SNPs and 1 InDel. It is possible to obtain accuracies of 0.92 AUC for red, 0.83 AUC for black, 0.80 AUC for blond and 0.72 AUC for brown. Moreover, the final HIrisPlex-S system can predict 5 skin pigmentation categories together with eye and hair colour using 41 SNPs with an accuracy of 0.74 AUC for very light, 0.72 AUC for light, 0.73 AUC for intermediate, 0.88 AUC for dark and 0.96 AUC for dark to black categories.

Other panels, more precisely the **SHEP** panels [123–125] to infer pigmentation traits, have been included in the **Snipper App** [73]. Regarding eye colour, this webtool allows to select between 7, 13 or 23 SNPs to infer blue, green/hazel, or brown eyes. Hair colour can be classified in two or four categories (light *vs* dark, or red *vs* blond *vs* brown *vs* black) when genotyping 12 markers, whereas skin colour is categorized as light, intermediate, or black typing 10 SNPs. These traits can be predicted using NB, MLR, or GDA.

### BGA and EVC

As observed, some physical characteristics, in particular pigmentation traits, vary according to continental populations [8, 11, 66, 80]. Thus, it is important to always consider both when interpretating the results. A total number of 15 panels that infer simultaneously BGA and EVC have been included in this review (Table 4).

**Table 4:** Commercial and non-commercial bio-geographical ancestry (BGA) and externally visible characteristics (EVC) panels proposed in the literature, including their reference article, number, and SNPs type, first used genotyping technology, prediction model, and inferred populations and traits.

	**SNPs**	**Genotyping technology**	**Statistical model**	**Inferred BGA and traits**	**Related articles**
MiSeq FGx™ Forensic Genomic System (includes ForenSeq™ Signature Kit B)	22 PISNPs 56 aAISNPs	NGS	Illumina ForenSeq Universal analysis Software™ (Undisclosed)	BGA (Undisclosed) Eye, hair, and skin colour	[28, 148, 180, 191, 192, 215, 304–318]
Parabon® Snapshot®	Undisclosed	NGS	Undisclosed	BGA (EUR, MED, EAS, CAS, AFR) Eye, hair, and skin colour Freckling Face shape	NA
[319]	6 PISNPs 4 AISNPs	SNaPshot™ + CE	NB (STRUCTURE)	BGA (EUR, AFR, ASN) Eye, hair, and skin colour	NA
[320]	60 PISNPs 43 AISNPs	SNaPshot™ + CE	NB (STRUCTURE)	BGA (AFR, AFR-AME, EUR, SAS, ASN, NAM, HIS) Eye, hair, and skin colour Hair morphology Male-pattern baldness	NA
[321]	21 mt-AISNPs 28 Y-AISNPs 14 AI-/PISNPs	SNaPshot™ + CE	GDA	BGA (AFR, EUR, NAF/MED, ASN, EAS) Eye, hair, and skin colour	NA
Identitas v1 Forensic Chip [128]	192 658 aSNPs 3 012 Y-SNPs 5 075 X-SNPs 428 mt-SNPs	Microchip	MLR	BGA (EUR, AFR, EAS, SAS, NAM) Eye and hair colour Kinship Sex	NA
[322]	31 PISNPs 19 AISNPs	SNaPshot™ + CE	MLR NB (Snipper)	BGA (EUR, AFR-AME, NAM/HIS, ASN) Eye colour	NA
32-plex [205, 323]	10 PISNPs 22 AISNPs	SNaPshot™ + CE	NB (STRUCTURE and Snipper) DAPC	BGA (AFR, EUR, SAS, EAS, NAM) Eye, hair, and skin colour	[246]
MiniPlex [158]	5 mt-AISNPs 4 Y-AISNPs 1 Y-AI InDel 5 aAISNPs 3 PISNPs	SNaPshot™ + CE	MLR, NB (Snipper)	BGA (5 global mt- and Y-haplogroups, AFR, EUR, EAS, OCE, NAM) Eye colour Lineage	NA
VISAGE Basic Tool for Ancestry and Appearance (BT A&A) [126]	41 PISNPs 153 AISNPs	NGS	NB (Snipper)	BGA (AFR, EUR, EAS, NAM, OCE, SAS) Eye, hair, and skin colour	[324–326]
Ion AmpliSeq™ PhenoTrivium Panel [327]	41 PISNPs 163 AISNPs 120 Y-AISNPs	NGS	NB (Snipper)	BGA (AFR, EAS, SAS, SWAS, EUR, NAM, OCE) Eye, hair, and skin colour	[197]
[328]	67 AISNPs 23 PISNPs 35 Y-AISNPs	NGS	NB (Snipper) CRT	BGA (Pakistan pub-populations) Eye, hair, and skin colour	NA
[203]	2 AISNPs 3 PISNPs	TaqMan® SNP genotyping	BN	BGA (EUR, ASN) Eye colour	NA
[240]	41 PISNPs 141 AISNPs	NGS	LR MLR	BGA (AFR, EUR, ASN, NAM, SWAS, MED) Eye, hair, and skin colour	NA
Phenotype Expert [329]	41 PISNPs 14 Y-ASNPs Amelogenin 4 ABO blood group SNPs	Microchip	MLR CRT	BGA (Slavic Y-haplogroups) Eye, hair, and skin colour	NA

EUR: European; AFR: African (NAF: North, AFR-AME: American); ASN: Asian (EAS: East, CAS: Central, SAS: South, SWAS: Southwest); NAM: Native American; OCE: Oceanian; MED: Mediterranean; HIS: Hispanic; NB: Naive Bayes; GDA: genetic distance algorithm; MLR: multinomial linear regression; DAPC: discriminant principal components analysis; CRT: classification and regression tree; BN: Bayesian Networks; LR: likelihood ratio.

The VISAGE Consortium presented their first appearance and ancestry single assay, referred as **VISAGE Basic Tool (BT)** [126]. It consists of a total of 153 AISNPs for continental origin inference, most of them part of the EUROFORGEN Global AIM-SNPs ancestry panel [78], two SNPs from Kidd’s panel [71, 77] and 11 from the Precision ID Ancestry Panel [71], and the 41 SNPs from the HIrisPlex-S panel [122] for pigmentation inference.

In terms of commercial solutions, the **MiSeq FGx™ Forensic Genomics System** (which includes the ForenSeq™ DNA Signature Prep Kit and ForenSeq™ Universal Analysis Software) (Illumina S.A., USA) [127] is one of the most complete forensic tools since it contains two panels: the first one including 27 autosomal, 7 X- and 24 Y-chromosomal STRs and 94 identity-SNPs for identification purposes, while the second panel contains 56 ancestry- (to classify four populations (European, American, African, and East Asian)) and 22 phenotype-informative SNPs (for eye and hair colour). Moreover, the VisiGen Consortium developed another commercial solution, which included **Identitas v1 Forensic Chip** and **Identify software** [128, 129], which allows inference of bi-parental BGA, eye and hair colour, relatedness, and sex by interrogating 201 173 genome-wide autosomal (192 658), Y- (3 012), X- (5 075) and mt-SNPs (428). Finally, Parabon Nanolabs offers the **Snapshot™ DNA Phenotyping Service** [130], which they deem capable of creating a complete profile, including genetic ancestry, eye, hair, and skin colour, freckling and face shape.

## Findings

The relatively new appearance of FDP and its debated implementation [131–135] translates into a complicated harmonization of its methodology, which is clear after inspecting all the SNP panels included in this review. It can be concluded that the factors that will influence the accuracy of the prediction are the genetic heritability of the trait, the method of SNP selection and genotyping, the informativeness of the SNP, the reference dataset, and the mathematical approach [7, 9, 12, 136]. Thus, before FDP methods can be used in forensic investigations, they need to be standardized and forensically validated, according to the Scientific Working Group on DNA Analysis Methods (SWGDAM) guidelines [137], to finally provide reliable and reproducible results. To do so, all the technical advantages and limitations of FDP must be considered. In addition, a consensus between researchers and field experts is needed to prepare protocols and directives to meet all ethical, social, and legal requirements (reviewed in [138]).

### Terminology and reporting

The first most important issue is the terminology employed to identify FDP research. Although the word “FDP” was already introduced in 2008 [10, 47], not all articles on BGA or EVC inference identify it as such and simply refer it as an intelligence tool. For instance, only 78 articles included in this review identify FDP (two of them as molecular photofitting). Thus, the correct identification of the term as keyword and in the text would allow a more congruent literature search.

Similarly, the second issue is the definition, categorization, and measurement of traits. On one hand, considering the nature of the traits (i.e. quantitative, like height and BMI; or qualitative, such as pigmentation traits and BGA), encasing the latter into categories, may lead to oversimplification [24], irreproducible results, and incomparable studies [128]. This especially becomes challenging when analysing data from multiple sources. Moreover, these categories are usually mistaken with stereotypes or sense of nationality [33, 128]. Although categorization in forensics is preferred [39, 139–141]—since the application in casework implies human interpretation (i.e. investigators)—, some researchers recommend using a continuous and quantitative spectrum instead [9, 142, 143]. On the other hand, measurements tend to be quite subjective, with most studies based on self-reported EVCs data *via* questionnaires or reported by simple observation of a non- or medical expert. For example, even when pigmentation traits are usually recorded *via* digital photographs they are later interpreted and put into categories by researchers. To avoid errors due to different perceptions of a trait [24, 144], several studies suggested applying specialized equipment and reflectance, bioimaging and biochemical technologies [145, 146] to find stronger genotype–phenotype associations [140]. In the case of BGA, information on up until a third-degree familial ancestry is usually reported and accompanied with a family pedigree.

The same issue arises when FDP results are being reported. For instance, Atwood et al. [34] compared different service providers in terms of prediction accuracy, clarity of reporting and consistent terminology, limitations, cost, and time. The authors concluded that it is imperative that guidelines are created for a shared methodology, and clear reporting and easy interpretation of the analysis for non-experts. Interestingly, results were shown in many ways, from simple verbal “not−/likely” to highlighting —or not— the highest probability for each trait variation or ancestry, or finally with a visual map representing where the individual falls on the represented population clusters.

### Development of panels

Before developing a panel for a certain trait or combinations of traits, researchers concentrate on finding the most informative set of markers for each trait. Usually, the discovery is performed *via* GWAS and later, confirmed by association studies [9, 13, 128]. This allows to avoid false positives and to find genes with weaker effects that may have been ignored [10, 14]. Even so, these studies are usually carried out with small sample size and are not extensively replicated, creating some scepticism on the validity of the found associations [14]. Ideally, a worldwide population scan would be key to find candidate genes [147], considering that normally sub-populations are less represented in exploratory panels [148]. Other studies find SNPs by comparing allele frequencies found in genetic population databases (e.g. HapMap, 1000 Genomes, CEPH Human Genome Diversity Panel, Complete Genomics) with specialized tools (e.g. SPSmart, FROG-kb [149, 150]).

In the case of BGA inference, it is important to select those variants with extreme allele frequency differences between populations [65, 69, 72, 151, 152] and obtain marker combinations to have equivalent levels of differentiation among those [78]. On the contrary, the genetic complexity of EVCs, due to pleiotropies (i.e. a single SNP influencing multiple traits) [11, 14], epistasis (i.e. several SNPs influencing a single trait) [11, 144, 153], allelic heterogeneity [83, 154, 155], phenotypic variability, and gene–environment interactions, need to be assessed before selecting the candidate markers. However, these genetic mechanisms are still not fully understood [9], and it is possible that many other implicated and more informative genes are being ignored [15].

One of the first debates is centred around the number of SNPs needed in a panel to obtain reliable predictions. On one hand, small SNP panels must contain the most informative and differentiating markers and are ideal for the current available SNaPshot™ technologies and to obtain lesser partial profiles when typing low DNA samples [156–158]. On the other side, increasing the number of SNPs improves the accuracy, especially with missing data [128, 152, 159, 160]. However, the number of SNPs will also depend on the analysis’ purpose and the genetic complexity of the trait. For instance, the four or five main continental populations can be distinguished with ease using less than 40 markers [39, 158], and eye colour can be distinguished with only six SNPs [83]. Conversely, even though the heritability of height and eye pigmentation is similar, the number of SNPs needed to infer stature is increasing by hundreds as its molecular mechanisms are discovered [161, 162]. In this sense, several authors believe that it is better to have markers with a strong influence [147, 163], due to the scare amount of DNA in the samples, while others suggest finding genes with weak effects to complement the inference [164–166]. Also, in the case of BGA, researchers recommend using a two-tier approach: first, a panel with maximum 100 markers to infer at least 12 global populations, and later other panels to refine sub-population inference [39, 58, 167, 168]. That is the case of the SNPforID 34-plex [156] and its Eurasiaplex [74], Pacifiplex [75], and PIMA’s [76] sub-panels.

Even though some researchers evaluated the capacity of EVC-associated variants to be used as AISNPs [97–99, 152, 163, 169–172], making indirect inferences based on either BGA or EVC is a highly debated practice. Indeed, some authors made assumptions about individuals’ appearance using only BGA data [34, 173], or *vice-versa* [16, 36, 67, 80, 174]. Nonetheless, most researchers discourage this practice, especially with the increasing population admixture [9, 33, 43, 147] and the fact that some shared alleles may not be related to ancestry but to environmental exposures that are the same in different populations [33]. Despite this, it is still important to infer BGA, as well as biological age and sex, together with EVCs, especially if a trait is restricted to a population, sex, or age group [10, 97, 175], to avoid any misleading interpretations.

Extensive lists of markers associated with EVCs are available [8, 15, 54] and they have been combined in multiple ways, yet the number of overlapping of unique markers is minimal. Soundararajan et al. [39] reported this same fact on BGA panels and emphasized the need for a collaboration among researchers to find the “best” markers and test them on a large data set representative of all global populations. Therefore, validation and inter-laboratory testing of panels is important to meet the specific quality requirements typical of forensic DNA analysis. Only few systems have been validated for forensic use [6, 7, 9, 11, 27]. Furthermore, panels are commonly developed using homogeneous and European reference data, and then validated in other populations; and they are replicated and validated by adopting different methodologies, generating a more complicated comparison exercise [39]. The best outcomes would be to adapt the panel to each individual population [176] or obtain a complete allele frequency data for all existing populations and subpopulations [168].

Another factor that influences this choice is if SNPs are found in “coding” or “non-coding” genes, and their informativeness of other health-related phenotypes. This first differentiation follows the legal regulations that have been used for STR identification and although scientists have discussed that these categories do not reflect the reality, it is still used as a reason to include or discard markers. However, FDP implies the use of “associative” markers that can be found in both non- and coding regions. The fear of including coding markers is based on their higher potential to provide health information [9, 80, 144], although non-coding genes can provide similar information if they are in linkage disequilibrium with the implicated coding genes [10] or regulatory regions [177]. Moreover, many disease- or trait-related candidate genes are first discovered when researching pathological or extreme variations, and other mutation within are found to be associated with normal variation instead [15, 142]. For example, *OCA2* gene mutations are associated with eye colour and oculocutaneous albinism [15, 24, 96]. Regarding these off-target phenotypes, Bradbury et al. [178] studied the possibility to reveal health information while predicting EVC, and only 27 out of 1 766 FDP-related markers were associated with risk of having cancer, induced asthma or risk of alcoholism. However, these associations do not mean that an individual is suffering from these diseases and a single marker cannot be used to predict or confirm these risks.

Finally, there is a continuous debate on using commercial or non-commercial panels. While commercial houses’ strongest point is their constant supply of ready-to-use kits, they claim the kit’s technical information (e.g. markers, accuracy, statistical model, etc.) as their intellectual property. Hence, researchers cannot ensure a truthful validation and reproducibility of the kit. Consequently, some companies have been discontinued, like DNAPrint [33]; while others, such as Parabon Nanolabs have been criticized by many FDP-experts [53].

### Genotyping technology

All available SNP typing methodologies have already been evaluated for forensic application [18–20, 22, 27]. These techniques are known to be very versatile, allowing the combination of different chemical reactions, assay formats and detection methods [19, 20]. Then again, not all techniques are suitable as FDP faces similar problems to STR identification when analysing forensic samples (i.e. low quantity and degraded DNA and often mixtures). The selection of methodology will be based on its accuracy, multiplexing and automation capacity, high-throughput, cost, and time; as well as the purpose of the analysis (e.g. the number of traits and markers to be included).

A great number of genetic techniques have been used to infer BGA or EVCs [13, 40, 179–181]: PCR assays (e.g. PCR-RFLP [103], PCR-REBA [182], and most commonly TaqMan® SNP genotyping assay), microarrays (e.g. GeneChip™ [128, 151]), minisequencing (e.g. SNPlex™), matrix-assisted laser desorption/ionization—time-of-flight (MALDI-TOF) together with mass spectrometry (MS) detection (e.g. Sequenom® MassARRAY®) [183–185] and high-resolution melting (HRM) [65, 179, 181]. While some techniques like Sequenom® MassARRAY® or HRM do not reach the sensitivity requirements for forensic samples [181, 183, 186], others have been developed but discontinued, such as Genomelab™ SNPstream® [66, 88, 91, 140] and Genplex®. Nonetheless, the golden standard is still SNaPshot™ (SBE-CE assay) due to its robustness, simplicity, and efficiency, but more precisely because the instrument is already present in forensic laboratories and great efforts were invested in their standardization [2, 13, 40, 179, 181].

Despite this, SNaPshot™-CE has a higher risk of contamination and error, and more importantly is limited to analyse one single trait inferred with 30 to 40 markers at a time and hence, it cannot keep up with the increasing number of markers needed for FDP [128, 187–189]. For this reason, researchers are shifting to NGS techniques, in particular Ion Torrent™ (ThermoFisher Scientific) and Illumina® [41, 187, 190]. They allow higher throughput, multiplexing capacity and sequencing accuracy [15], as well as the possibility to automate and sequence different markers in the same run (e.g. STR, SNPs, InDels, microhaplotypes) [23, 191]. However, this implies a longer preparation, sequencing, and analysis time [192]. As a result, the current focus is on testing SNaPshot™ panels using NGS instruments [175, 187–189, 193, 194], applying single cell sequencing and NGS to analyse mixtures and touch DNA samples [160, 195–197], and automating analysis and result interpretation to reduce analysis time [23]. This last one would allow a better handling of the samples, increase the sample size, and reduce costs and time.

All these techniques have their advantages and limitations, making it harder to choose one to proceed with their standardization. Moreover, the methodology will be chosen depending on the investigation requirements and purpose [8, 14, 19, 187] and any new one such as massively parallel sequencing (MPS) needs to be extensively validated in larger datasets and optimized before being incorporated [35, 157, 192]. Other factors that restrain technological advancement in the field are the costs to renovate workspaces, to train the staff, and to increase bioinformatic support and storage capacity [13, 44, 45, 158].

### Prediction models and algorithms

Prediction models are created to support and understand the relationship between genotype and phenotype [14, 15]. There are two types of algorithms that can be used to predict BGA or EVC outcomes: statistical and machine-learning (ML). Statistical algorithms, such as MLR, work better when the predictors are dependent from each other, while ML algorithms usually assume independence among predictors [15] and detect in a linear or more complex way the dependency between variables and attributes [198]. Both methods may provide similar accuracy when the same SNP panel is used [15], although ML methods require a higher computational cost and expertise. Indeed, several articles compared and introduced different classifiers for FDP analysis [12, 48, 67, 68, 70, 139, 198–204].

Two of the most used programmes, STRUCTURE and Snipper, are based on the NB algorithm. This algorithm calculates how likely a trait belongs to a class comparing it with the allele frequencies that are observed in each cluster and make assumptions on unknown profiles [68, 156]. It is also capable of incorporating missing data [68]. The gold standard for BGA inference is the STRUCTURE software (and its updated version, ADMIXTURE), because of its “efficient clustering based on similarities or dissimilarities with the other samples” [48, 49, 78] and, thus, good inference of admixture proportions, but only if the populations are well differentiated in the reference data [156, 199]. Its main disadvantages are assuming HWE, which is not compatible with BGA nor EVC inference [68], and its long and computationally intensive run times when classifying single profiles with large datasets—since the parental data and the unknown profile need to be analysed simultaneously and missing data needs to be imputed. Otherwise, Snipper can solve some of the issues STRUCTURE presents, providing a faster analysis [72, 156], allowing the incorporation of one’s own reference dataset and being able to classify single profiles in real time [74]. The later has been both used for BGA and EVC inference.

Other alternative methods have also been tested. For example, GDA provides a continuous clustering by evaluating the informative proportions of each component, it does not assume HWE, and it can be used as input for hierarchical clustering, like neighbour joining trees. Although it is highly sensitive to noise [48], it has been proven better for admixture classification [67, 199]. On another note, visual representations of individual and population structure like principal component analysis, discriminant analysis of principal components [205], or multidimensional scaling are helpful to interpretate the outcome [68]. However, since they are reduced to the two or three most important components, it may lead to misclassification [48]. In addition, logistic regression (bi- or multinomial likelihood ratio, LR) is perfect for assessing categorical outcomes, even though it tends to misclassify partial profiles [68]. It has been traditionally applied to infer pigmentation colours [83, 120]. Also, multifactor dimensionality reduction is used in small sample size studies to better detect epistatic effects [124, 153, 206]. Other available and tested ML methods are linear discriminant analysis, support vector machine [163, 166, 198], partial least square regression [88], extreme gradient boosting [198, 207], classification and regression trees [164, 198, 208, 209], multi-variate adaptive regression splines [198], bootstrapped response-based imputation modelling, ordinal and stepwise regressions [207, 210], and deep learning approaches such as neural networks (NN) and Random Forest [67, 87, 115, 166, 198, 199, 202, 207]. NN are proposed as an alternative to LR as it recognizes the patterns of complex data typical from EVC inference [88, 164].

Hence, not all algorithms are appropriate, and will need to be selected depending on several aspects. First, the amount and type of data [198], as well as the impact of missing/partial profiles in the classification performance [68, 72]. Second, the reference population, which not only affects the selection of SNPs but also the training of the classifiers. These must be representative of all variations and ancestries, especially when estimating admixed individuals [35, 67, 68, 199, 211]. Third, with the inability to incorporate environmental factors to the prediction, only sex and age can be incorporated as covariates. In the same way, the accuracy of the model will increase when considering both BGA and EVC if there is population dependency [142, 212]. Some researchers defend that “when all the causing factors of a trait will be accounted for in the model, then the accuracy will be the same in all populations” [213].

Lastly, there are many options to interpretate the results obtained from the prediction model. It is key that field and legal experts easily understand and apply the findings. Logically, one may recommend continuing using LR, since it already used in STR identification [142, 143, 214, 215]. Nonetheless, as Caliebe et al. [176] observed, since FDP does not apply the same principle of comparing two hypotheses (i.e. sample belonging to a random individual *vs* the suspect), and the highest value may not represent the correct category [35]. Hence, it will be more appropriate to use statistical probability, represented as posterior odds, but unfortunately, statistics are often harder to understand by the plain audience. Other ways to represent accuracy have been incorporated: AUC for categorical predictions—that vary from 0.5 (random phenotype) to 1 (exact phenotype) [7, 11, 12, 15, 17]; and correlation (*R* or *R*^2^) or mean squared error (MSE) for quantitative measurements [15].

## Conclusions, limitations, and recommendations

The expectations that the forensic experts have on FDP reveals the need to provide accurate and tangible results to solve more complicated investigations. In this review, we investigated those panels that had been developed precisely for FDP and analysed the limitations to have in mind before an agreed application of the technique in the forensic workload. Among the available bibliography, 304 publications were strictly related to FDP inference and only 80 of them clearly identified that the research was for FDP inference. A total of 48 panels have been developed for BGA inference, six being commercial tests; while only one of the 43 panels available to infer several EVCs is from a commercial vendor. In addition, BGA and EVC can be simultaneously inferred with 15 panels, two of which are wildly used commercial solutions.

Throughout the literature, there is a recurrent stance from researchers: FDP is not to use in trial, but during the investigation step. This reasoning is because FDP cannot reach the level of “scientific certainty” that has been attributed to STR identification. Hence, although the justice seeks for an “absolute truth”, there needs to be a shift regarding the expectations on the results’ conclusiveness [2]. Realistically, in the near future of FDP, accuracy will not improve drastically. This is because even if more genetic and environmental interactions are found, the fully understanding of the effects on phenotypes complicates at the same time. There are a few things that can be done to increase the results accuracy, such as using quantitative and continuous predictions, promoting validation on all possible human populations and sub-populations, and investigating the incorporation of prior knowledge in the models [200]. The same can be said about incorporating other traits into the FDP profile, since the current extensive research (on height, weight, and facial morphology, among others) does not provide enough weight to obtain acceptable prediction accuracies. Moreover, there is an increasing interest in combining FDP with epigenetic information, not only to infer age, but to infer traits that are age-dependent like hair greying, and with other types of analysis, such as investigative genetic genealogy or behavioural tendencies. These last two come with many ethical implications, such as the violation of genetic information of family members or whether a tendency such as aggression or depression is more influenced by physiological, rather than genetic factors and, thus, considered medical information.

Finally, a decision concerning methodology advancement must be made by forensic services, either MPS is incorporated to laboratories to keep up with the increasing demand of high number of markers and traits—that current SNaPshot™ methods cannot, or either, if FDP is considered as a tool that will not be used regularly and only in “desperate times”, this task is to be entrusted to specialized external centres. Nonetheless, the advancement of FDP application will rest on the efforts of the forensic community on creating guidelines and standards for EVC and BGA inference, from their measurement and categorization to their genotyping and prediction models.

## Authors’ contributions

Nuria Terrado-Ortuño carried out the conceptualization and drafted the manuscript. Patrick May participated in its design and critical review of this manuscript. Both authors contributed to the final text and approved it.

## Compliance with ethical standard

The opinions expressed in this paper belong to the authors and do not necessarily reflect the opinion of PCI. No human participants were involved in this review paper.

## Disclosure statement

None to declare.

## Funding

This work was supported by the Institute of Advanced Studies (University of Luxembourg) under an Audacity grant (AUDACITY-2020): Meet the Unknown: The Future of CRIMinal Forensic Genomics PhenoTYPing (CRIMTYP).

## Supplementary Material

FDP-panels-techniques-and-prediction-models_Suppl_Material_owae013

## References

[1] Jobling MA, Gill P. Encoded evidence: DNA in forensic analysis. Nat Rev Genet. 2004;5:739–751. 15510165 10.1038/nrg1455

[2] Walsh SJ . Recent advances in forensic genetics. Expert Rev Mol Diagn. 2004;4:31–40. 14711347 10.1586/14737159.4.1.31

[3] Daniel R, Walsh SJ. The continuing evolution of forensic DNA profiling—from STRs to SNPs. Aust J Forensic Sci. 2006;38:59–74.

[4] Butler JM, Coble MD, Vallone PM. STRs *vs*. SNPs: thoughts on the future of forensic DNA testing. Forensic Sci Med Pathol. 2007;3:200–205. 25869164 10.1007/s12024-007-0018-1

[5] Jobling MA . Y-chromosomal SNP haplotype diversity in forensic analysis. Forensic Sci Int. 2001;118:158–162. 11311830 10.1016/s0379-0738(01)00385-1

[6] Matheson S . DNA phenotyping: snapshot of a criminal. Cell. 2016;166:1061–1064. 27565333 10.1016/j.cell.2016.08.016

[7] Schneider PM, Prainsack B, Kayser M. The use of forensic DNA phenotyping in predicting appearance and biogeographic ancestry. Dtsch Arztebl Int. 2019;116:873. 10.3238/arztebl.2019.0873 PMC6976916 31941575

[8] Tozzo P, Politi C, Delicati A, et al. External visible characteristics prediction through SNPs analysis in the forensic setting: a review. Front Biosci (Landmark Ed). 2021;26:828–850. 34719209 10.52586/4991

[9] Kayser M . Forensic DNA phenotyping: DNA testing for externally visible characteristics. In: Siegel JA, Saukko PJ, Houck MM, editors. Encyclopedia of Forensic Sciences. 2nd ed. Waltham (MA): Academic Press; 2013. p. 369–374.

[10] Kayser M, Schneider PM. DNA-based prediction of human externally visible characteristics in forensics: motivations, scientific challenges, and ethical considerations. Forensic Sci Int Genet. 2009;3:154–161. 19414162 10.1016/j.fsigen.2009.01.012

[11] Canales SA . [Forensic DNA phenotyping: a promising tool to aid forensic investigation. Current situation]. J Leg Med. 2020;46:183–190. Spanish.

[12] Pośpiech E, Teisseyre P, Mielniczuk J, et al. Predicting physical appearance from DNA data: towards genomic solutions. Genes. 2022;13:121. 35052461 10.3390/genes13010121 PMC8774670

[13] Mehta B, Daniel R, Phillips C, et al. Forensically relevant SNaPshot® assays for human DNA SNP analysis: a review. Int J Leg Med. 2016;131:21–37. 10.1007/s00414-016-1490-5 27841004

[14] Pulker H, Lareu MV, Phillips C, et al. Finding genes that underlie physical traits of forensic interest using genetic tools. Forensic Sci Int Genet. 2007;1:100–104. 19083737 10.1016/j.fsigen.2007.02.009

[15] Kayser M, Forensic DNA. Phenotyping: predicting human appearance from crime scene material for investigative purposes. Forensic Sci Int Genet. 2015;18:33–48. 25716572 10.1016/j.fsigen.2015.02.003

[16] Stephan CN, Caple JM, Guyomarc’h P, et al. An overview of the latest developments in facial imaging. Forensic Sci Res. 2019;4:10–28. 30915414 10.1080/20961790.2018.1519892 PMC6427692

[17] Haddrill PR . Developments in forensic DNA analysis. Emerg Top Life Sci. 2021;5:381–393. 33792660 10.1042/ETLS20200304 PMC8457771

[18] Budowle B . SNP typing strategies. Forensic Sci Int. 2004;146:S139–S142. 15724292 10.1016/j.forsciint.2004.09.042

[19] Sobrino B, Carracedo A. SNP typing in forensic genetics: a review. Methods Mol Biol. 2005;297:107–126. 15570103 10.1385/1-59259-867-6:107

[20] Sobrino B, Brión M, Carracedo A. SNPs in forensic genetics: a review on SNP typing methodologies. Forensic Sci Int. 2005;154:181–194. 16182964 10.1016/j.forsciint.2004.10.020

[21] Decorte R . Genetic identification in the 21st century—current status and future developments. Forensic Sci Int. 2010;201:160–164. 20347537 10.1016/j.forsciint.2010.02.029

[22] Ziętkiewicz E, Witt M, Daca P, et al. Current genetic methodologies in the identification of disaster victims and in forensic analysis. J Appl Genet. 2011;53:41–60. 22002120 10.1007/s13353-011-0068-7 PMC3265735

[23] Kowalczyk M, Zawadzka E, Szewczuk D, et al. Molecular markers used in forensic genetics. Med Sci Law 2018; 58:201–209. 30269675 10.1177/0025802418803852

[24] Liu F, Wen B, Kayser M. Colorful DNA polymorphisms in humans. Semin Cell Dev Biol. 2013;24:562–575. 23587773 10.1016/j.semcdb.2013.03.013

[25] Gurkan C, Bulbul O, Kidd KK. Editorial: current and emerging trends in human identification and molecular anthropology. Front Genet. 2021;12:1041. 10.3389/fgene.2021.708222 PMC8262838 34249111

[26] Oh CS, Shin DH, Hong JH, et al. Single-nucleotide polymorphism analyses on ABCC11, EDAR, FGFR2, and ABO genotypes of mummified people of the Joseon Dynasty, South Korea. Anthropol Sci. 2018;126:67–73.

[27] Watherston J, McNevin D, Gahan ME, et al. Current and emerging tools for the recovery of genetic information from post mortem samples: new directions for disaster victim identification. Forensic Sci Int Genet. 2018;37:270–282. 30181101 10.1016/j.fsigen.2018.08.016

[28] Ambers A, Bus MM, King JL, et al. Forensic genetic investigation of human skeletal remains recovered from the La Belle shipwreck. Forensic Sci Int. 2020;306:110050. 31790892 10.1016/j.forsciint.2019.110050

[29] Bogdanowicz W, Allen M, Branicki W, et al. Genetic identification of putative remains of the famous astronomer Nicolaus Copernicus. Proc Natl Acad Sci U S A. 2009;106:12279–12282. 19584252 10.1073/pnas.0901848106 PMC2718376

[30] Kupiec T, Branicki W. Genetic examination of the putative skull of Jan Kochanowski reveals its female sex. Croat Med J. 2011;52:403–409. 21674838 10.3325/cmj.2011.52.403 PMC3118727

[31] King TE, Fortes GG, Balaresque P, et al. Identification of the remains of King Richard III. Nat Commun. 2014;5:1–8. 10.1038/ncomms6631 PMC4268703 25463651

[32] Zupanič PI . Identification of a Slovenian pre-war elite couple killed in the Second World War. Forensic Sci Int. 2021;327:110994. 34536754 10.1016/j.forsciint.2021.110994

[33] Sankar P . Forensic DNA phenotyping: continuity and change in the history of race, genetics, and policing. In: Wailoo K, Nelson A, Lee C, editors. Genetics and the unsettled past: the collision of DNA, race, and history. New Brunswick (NJ): Rutgers University Press; 2012. p. 104–113.

[34] Atwood L, Raymond J, Sears A, et al. From identification to intelligence: an assessment of the suitability of forensic DNA phenotyping service providers for use in Australian law enforcement casework. Front Genet. 2021;11:1–11. 10.3389/fgene.2020.568701 PMC7835938 33510767

[35] Hollard C, Keyser C, Delabarde T, et al. Case report: on the use of the HID-ion AmpliSeqTM ancestry panel in a real forensic case. Int J Leg Med. 2017;131:351–358. 10.1007/s00414-016-1425-1 27470319

[36] Graham EAM . DNA reviews: predicting phenotype. Forensic Sci Med Pathol. 2008;4:196–199. 19291462 10.1007/s12024-008-9056-6

[37] Lippert C, Sabatini R, Maher MC, et al. Identification of individuals by trait prediction using whole-genome sequencing data. Proc Natl Acad Sci. 2017;114:10166–10171. 28874526 10.1073/pnas.1711125114 PMC5617305

[38] Butler JM, Reeder DJ. STRBase. USA: National Institute of Standards and Technology. Available from: https://strbase-archive.nist.gov/

[39] Soundararajan U, Yun L, Shi M, et al. Minimal SNP overlap among multiple panels of ancestry informative markers argues for more international collaboration. Forensic Sci Int Genet. 2016;23:25–32. 26977931 10.1016/j.fsigen.2016.01.013

[40] Mehta BM . Genotyping Tools for Forensic DNA Phenotyping: From Low- to High-Throughput [dissertation]. Canberra (Australia): University of Canberra; 2019.

[41] Børsting C, Morling N. Next generation sequencing and its applications in forensic genetics. Forensic Sci Int Genet. 2015;18:78–89. 25704953 10.1016/j.fsigen.2015.02.002

[42] Budowle B, van Daal A. Forensically relevant SNP classes. Biotechniques. 2008;44:603–610. 18474034 10.2144/000112806

[43] Kayser M, de Knijff P. Improving human forensics through advances in genetics, genomics and molecular biology. Nat Rev Genet. 2011;12:179–192. 21331090 10.1038/nrg2952

[44] Wells JD, Linville JG. Biology/DNA/Entomology: Overview. In: Siegel JA, Saukko PJ, Houck MM, editors. Encyclopedia of Forensic Sciences. 2nd ed. Waltham (MA): Academic Press; 2013. p. 387–393.

[45] Morelato M, Barash M, Blanes L, et al. Forensic science: current state and perspective by a group of early career researchers. Found Sci. 2016;22:799–825.

[46] Pope S, Puch-Solis R. Interpretation of DNA data within the context of UK forensic science — investigation. Emerg Top Life Sci. 2021;5:395–404. 34151948 10.1042/ETLS20210165 PMC8457768

[47] Frudakis TN . Molecular Photofitting: Predicting Ancestry and Phenotype Using DNA. Cambridge (MA): Academic Press Inc.; 2007.

[48] Cheung EYY, Gahan ME, McNevin D. Predictive DNA analysis for biogeographical ancestry. Aust J Forensic Sci. 2018;50:1–8.

[49] Phillips C . Forensic genetic analysis of bio-geographical ancestry. Forensic Sci Int Genet. 2015;18:49–65. 26013312 10.1016/j.fsigen.2015.05.012

[50] Tully G . Genotype versus phenotype: human pigmentation. Forensic Sci Int Genet. 2007;1:105–110. 19083738 10.1016/j.fsigen.2007.01.005

[51] Branicki W . Studies on predicting pigmentation phenotype for forensic purposes. Probl Forensic Sci. 2009;77:29–52.

[52] Branicki W, Kayser M. Prediction of human pigmentation traits from DNA polymorphisms. eLS. 2015;1:1–10.

[53] Wolinsky H . CSI on steroids: DNA-based phenotyping is helping police derive visual information from crime scene samples to aid in the hunt for suspects. EMBO Rep. 2015;16:782–786. 26077709 10.15252/embr.201540714 PMC4515117

[54] Dabas P, Jain S, Khajuria H, et al. Forensic DNA phenotyping: inferring phenotypic traits from crime scene DNA. J Forensic Leg Med. 2022;88:102351. 35427851 10.1016/j.jflm.2022.102351

[55] Tricco AC, Lillie E, Zarin W, et al. PRISMA extension for Scoping Reviews (PRISMA-ScR): checklist and explanation. Ann Intern Med. 2018;169:467–473. 30178033 10.7326/M18-0850

[56] Kayser M . In reply. Dtsch Arztebl Int. 2020;117:269–270. 10.3238/arztebl.2020.0269b PMC7268094 32449893

[57] Al-Asfi M, McNevin D, Mehta B, et al. Assessment of the Precision ID Ancestry Panel. Int J Leg Med. 2018;132:1581–1594. 10.1007/s00414-018-1785-9 29556719

[58] Bulbul O, Speed WC, Gurkan C, et al. Improving ancestry distinctions among Southwest Asian populations. Forensic Sci Int Genet. 2018;35:14–20. 29625264 10.1016/j.fsigen.2018.03.010

[59] Lowe AL, Urquhart A, Foreman LA, et al. Inferring ethnic origin by means of an STR profile. Forensic Sci Int. 2001;119:17–22. 11348789 10.1016/s0379-0738(00)00387-x

[60] Rowold DJ, Herrera RJ. Inferring recent human phylogenies using forensic STR technology. Forensic Sci Int. 2003;133:260–265. 12787663 10.1016/s0379-0738(03)00073-2

[61] Phillips C, Santos C, Fondevila M, et al. Inference of ancestry in forensic analysis I: autosomal ancestry-informative marker sets. Methods Mol Biol. 2016;1420:233–253. 27259744 10.1007/978-1-4939-3597-0_18

[62] Oldoni F, Kidd KK, Podini D. Microhaplotypes in forensic genetics. Forensic Sci Int Genet. 2019;38:54–69. 30347322 10.1016/j.fsigen.2018.09.009

[63] Kayser M . Forensic use of Y-chromosome DNA: a general overview. Hum Genet. 2017;136:621–635. 28315050 10.1007/s00439-017-1776-9 PMC5418305

[64] Kivisild T . Maternal ancestry and population history from whole mitochondrial genomes. Investigative Genet. 2015;6:3. 10.1186/s13323-015-0022-2 PMC4367903 25798216

[65] Prestes PR, Mitchell RJ, Daniel R, et al. Predicting biogeographical ancestry in admixed individuals—values and limitations of using uniparental and autosomal markers. Aust J Forensic Sci. 2015;48:10–23.

[66] Halder I, Shriver M, Thomas M, et al. A panel of ancestry informative markers for estimating individual biogeographical ancestry and admixture from four continents: utility and applications. Hum Mutat. 2008;29:648–658. 18286470 10.1002/humu.20695

[67] Qu Y, Tran D, Ma W. Deep learning approach to biogeographical ancestry inference. Procedia Comput Sci. 2019;159:552–561.

[68] Cheung EYY, Gahan ME, McNevin D. Prediction of biogeographical ancestry from genotype: a comparison of classifiers. Int J Leg Med. 2017;131:901–912. 10.1007/s00414-016-1504-3 27995319

[69] Parfenchyk MS, Kotava SA. The theoretical framework for the panels of DNA markers formation in the forensic determination of an individual ancestral origin. Russ J Genet. 2021;57:1–9.

[70] Alladio E, Poggiali B, Cosenza G, et al. Multivariate statistical approach and machine learning for the evaluation of biogeographical ancestry inference in the forensic field. Sci Rep. 2022;12:1–17. 35643723 10.1038/s41598-022-12903-0 PMC9148302

[71] Thermo Fisher Scientific. Precision ID Ancestry Panel. Last revision: 2022. Available from: https://www.thermofisher.com/order/catalog/product/A25642

[72] Phillips C, Salas A, Sánchez JJ, et al. Inferring ancestral origin using a single multiplex assay of ancestry-informative marker SNPs. Forensic Sci Int Genet. 2007;1:273–280. 19083773 10.1016/j.fsigen.2007.06.008

[73] Universidad de Santiago de Compostela. Snipper app suite version 3. Last revision: April 2024. Available from: http://mathgene.usc.es/snipper/

[74] Phillips C, Aradas AF, Kriegel AK, et al. Eurasiaplex: a forensic SNP assay for differentiating European and South Asian ancestries. Forensic Sci Int Genet. 2013;7:359–366. 23537756 10.1016/j.fsigen.2013.02.010

[75] Santos C, Phillips C, Fondevila M, et al. Pacifiplex: an ancestry-informative SNP panel centred on Australia and the Pacific region. Forensic Sci Int Genet. 2016;20:71–80. 26517174 10.1016/j.fsigen.2015.10.003

[76] Carvalho Gontijo C, Porras-Hurtado LG, Freire-Aradas A, et al. PIMA: a population informative multiplex for the Americas. Forensic Sci Int Genet. 2020;44:102200. 31760353 10.1016/j.fsigen.2019.102200

[77] Kidd KK, Speed WC, Pakstis AJ, et al. Progress toward an efficient panel of SNPs for ancestry inference. Forensic Sci Int Genet. 2014;10:23–32. 24508742 10.1016/j.fsigen.2014.01.002

[78] Phillips C, Parson W, Lundsberg B, et al. Building a forensic ancestry panel from the ground up: the EUROFORGEN Global AIM-SNP set. Forensic Sci Int Genet. 2014;11:13–25. 24631693 10.1016/j.fsigen.2014.02.012

[79] Pereira V, Freire-Aradas A, Ballard D, et al. Development and validation of the EUROFORGEN NAME (North African and Middle Eastern) ancestry panel. Forensic Sci Int Genet. 2019;42:260–267. 31404905 10.1016/j.fsigen.2019.06.010

[80] Branicki W, Brudnik U, Wojas-pelc A. Genetic prediction of pigmentary traits in forensic studies. Forensic Sci Int. 2005;64:343–357.

[81] Martinez-Cadenas C, Penãa-Chilet M, Ibarrola-Villava M, et al. Gender is a major factor explaining discrepancies in eye colour prediction based on HERC2/OCA2 genotype and the IrisPlex model. Forensic Sci Int Genet. 2013;7:453–460. 23601698 10.1016/j.fsigen.2013.03.007

[82] Pietroni C, Andersen JD, Johansen P, et al. The effect of gender on eye colour variation in European populations and an evaluation of the IrisPlex prediction model. Forensic Sci Int Genet. 2014; 11:1–6. 10.1016/j.fsigen.2014.02.002 24631691

[83] Walsh S, Liu F, Ballantyne KN, et al. IrisPlex: a sensitive DNA tool for accurate prediction of blue and brown eye colour in the absence of ancestry information. Forensic Sci Int Genet. 2011;5:170–180. 20457092 10.1016/j.fsigen.2010.02.004

[84] Gupta V, Sharma VK. Skin typing: Fitzpatrick grading and others. Clin Dermatol. 2019;37:430–436. 31896400 10.1016/j.clindermatol.2019.07.010

[85] Kukla-Bartoszek M, Pośpiech E, Woźniak A, et al. DNA-based predictive models for the presence of freckles. Forensic Sci Int Genet. 2019;42:252–259. 31400656 10.1016/j.fsigen.2019.07.012

[86] Hernando B, Ibañez MV, Deserio-Cuesta JA, et al. Genetic determinants of freckle occurrence in the Spanish population: towards ephelides prediction from human DNA samples. Forensic Sci Int Genet. 2018;33:38–47. 29190509 10.1016/j.fsigen.2017.11.013

[87] Cho S, Lee EH, Kim H, et al. Validation of BMI genetic risk score and DNA methylation in a Korean population. Int J Leg Med. 2021;135:1201–1212. 10.1007/s00414-021-02517-y 33594455

[88] Claes P, Hill H, Shriver MD. Toward DNA-based facial composites: preliminary results and validation. Forensic Sci Int Genet. 2014;13:208–216. 25194685 10.1016/j.fsigen.2014.08.008

[89] Samuels BD, Aho R, Brinkley JF. et al. FaceBase 3: analytical tools and FAIR resources for craniofacial and dental research. Development. 2020;147:dev191213. 10.1242/dev.191213 PMC7522026 32958507

[90] The International Visible Trait Genetics (VisiGen) Consortium [Internet].

[91] Claes P, Liberton DK, Daniels K, et al. Modeling 3D facial shape from DNA. PLoS Genet. 2014;10:e1004224. 24651127 10.1371/journal.pgen.1004224 PMC3961191

[92] Phillips C, Barbaro A, Lareu MV, et al. Initial study of candidate genes on chromosome two for relative hand skill. Int Congr Ser. 2006;1288:798–800.

[93] van Daal A . The genetic basis of human pigmentation. Forensic Sci Int Genet Suppl Ser. 2008;1:541–543.

[94] Fridman C, Cardena MMSG, De A Lima F, et al. Is it possible to use forensic DNA phenotyping in Brazilian population? Forensic Sci Int Genet Suppl Ser. 2015;5:e378–e380.

[95] de Araújo LF, de Toledo GF, Fridman C. *SLC24A5* and *ASIP* as phenotypic predictors in Brazilian population for forensic purposes. Leg Med. 2015;17:261–266. 10.1016/j.legalmed.2015.03.001 25801600

[96] Andrade ES, Fracasso NCA, Strazza Júnior PS, et al. Associations of *OCA2-HERC2* SNPs and haplotypes with human pigmentation characteristics in the Brazilian population. Leg Med. 2017;24:78–83. 10.1016/j.legalmed.2016.12.003 28081795

[97] Veltre V, de Angelis F, Biondi G, et al. Evaluation of skin-related variants in African ancestry populations and their role in personal identification. Forensic Sci Int Genet Suppl Ser. 2019;7:172–174.

[98] Andersen JD, Meyer OS, Simão F, et al. Skin pigmentation and genetic variants in an admixed Brazilian population of primarily European ancestry. Int J Leg Med. 2020;134:1569–1579. 10.1007/s00414-020-02307-y 32385594

[99] Zaumsegel D, Rothschild MA, Schneider PM. SNPs for the analysis of human pigmentation genes—a comparative study. Forensic Sci Int Genet Suppl Ser. 2008;1:544–546.

[100] Branicki W, Szczerbińska A, Brudnik U, et al. The *OCA2* gene as a marker for eye colour prediction. Forensic Sci Int Genet Suppl Ser. 2008;1:536–537.

[101] Andersen JD, Johansen P, Wulf HC, et al. Genetic variants and skin colour in Danes. Forensic Sci Int Genet Suppl Ser. 2011;3:e153–e154.

[102] Andersen JD, Pietroni C, Johansen P, et al. Importance of nonsynonymous *OCA2* variants in human eye color prediction. Mol Genet Genomic Med. 2016;4:420–430. 27468418 10.1002/mgg3.213 PMC4947861

[103] de A Fracasso NC, de Andrade ES, CEV W, et al. Haplotypes from the *SLC45A2* gene are associated with the presence of freckles and eye, hair and skin pigmentation in Brazil. Leg Med. 2017;25:43–51. 10.1016/j.legalmed.2016.12.013 28457509

[104] Meyer OS, Lunn MMB, Garcia SL, et al. Association between brown eye colour in rs12913832:GG individuals and SNPs in *TYR*, *TYRP1*, and *SLC24A4*. PloS One. 2020;15:e0239131. 32915910 10.1371/journal.pone.0239131 PMC7485777

[105] Andersen JD, Johansen P, Mogensen HS, et al. Eye colour and SNPs in Danes. Forensic Sci Int Genet Suppl Ser. 2011;3:e151–e152.

[106] Salvo NM, Mathisen MG, Janssen K, et al. Experimental long-distance haplotyping of *OCA2-HERC2* variants. Forensic Sci Int Genet Suppl Ser. 2022;8:188–190.

[107] Yan J, Cao LP, Ye Y, et al. Association of melanocortin-1-receptor gene polymorphism with freckles in Chinese Han population. Forensic Sci Int Genet Suppl Ser. 2013;4:e320–e321.

[108] Fridman C, Ferreira MA, Marano LA, et al. Analysis of genetic polymorphisms associated with the presence of freckles for phenotypic prediction. Forensic Sci Int Genet Suppl Ser. 2022;8:26–28.

[109] Liu F, van der Lijn F, Schurmann C, et al. A genome-wide association study identifies five loci influencing facial morphology in Europeans. PLoS Genet. 2012;8:e1002932. 23028347 10.1371/journal.pgen.1002932 PMC3441666

[110] Wang Q, Jin B, Luo X, et al. Association between *BMP4* gene polymorphisms and eyelid traits in Chinese Han population. Forensic Sci Int Genet Suppl Ser. 2017;6:e355–e356.

[111] Li L, Wang Q, Wu S, et al. What makes your “eyes” look different? Forensic Sci Int Genet Suppl Ser. 2019;7:105–106.

[112] Jin B, Zhu J, Wang H, et al. A primary investigation on SNPs associated with eyelid traits of Chinese Han adults. Forensic Sci Int Genet Suppl Ser. 2015;5:e669–e670.

[113] Wang Q, Jin B, Liu F, et al. DNA-based eyelid trait prediction in Chinese Han population. Int J Leg Med. 2021;135:1743–1752. 10.1007/s00414-021-02570-7 33969445

[114] Xie M, Song F, Li J, et al. Characteristics of SNPs related with high myopia traits in Chinese Han population. Forensic Sci Int Genet Suppl Ser. 2017;6:e35–e36.

[115] Pośpiech E, Kukla-Bartoszek M, Karłowska-Pik J, et al. Exploring the possibility of predicting human head hair greying from DNA using whole-exome and targeted NGS data. BMC Genomics. 2020;21:1–18. 10.1186/s12864-020-06926-y PMC7430834 32758128

[116] Adhikari K, Fontanil T, Cal S, et al. A genome-wide association scan in admixed Latin Americans identifies loci influencing facial and scalp hair features. Nat Commun. 2016;7:1–12. 10.1038/ncomms10815 PMC4773514 26926045

[117] Pośpiech E, Chen Y, Kukla-Bartoszek M, et al. Towards broadening forensic DNA phenotyping beyond pigmentation: improving the prediction of head hair shape from DNA. Forensic Sci Int Genet. 2018;37:241–251. 30268682 10.1016/j.fsigen.2018.08.017

[118] Jawad M, Adnan A, Rehman RA, et al. Evaluation of facial hair-associated SNPs: a pilot study on male Pakistani Punjabi population. Forensic Sci Med Pathol. 2022;19:293–302. 35994154 10.1007/s12024-022-00515-z

[119] International Society of Genetic Genealogy Wiki. DNAPrint Genomics. Last revision: 2015. Available from: https://isogg.org/wiki/DNAPrint_Genomics

[120] Walsh S, Liu F, Wollstein A, et al. The HIrisPlex system for simultaneous prediction of hair and eye colour from DNA. Forensic Sci Int Genet. 2013;7:98–115. 22917817 10.1016/j.fsigen.2012.07.005

[121] Chaitanya L, Breslin K, Zuñiga S, et al. The HIrisPlex-S system for eye, hair and skin colour prediction from DNA: introduction and forensic developmental validation. Forensic Sci Int Genet. 2018;35:123–135. 29753263 10.1016/j.fsigen.2018.04.004

[122] Department of Genetic Identification of Erasmus MC. HIrisPlex-S Eye, Hair and Skin Colour DNA Phenotyping Webtool. Last revision: 2022. Available from: https://hirisplex.erasmusmc.nl/

[123] Ruiz Y, Phillips C, Gomez-Tato A, et al. Further development of forensic eye color predictive tests. Forensic Sci Int Genet. 2013;7:28–40. 22709892 10.1016/j.fsigen.2012.05.009

[124] Söchtig J, Phillips C, Maroñas O, et al. Exploration of SNP variants affecting hair colour prediction in Europeans. Int J Leg Med. 2015;129:963–975. 10.1007/s00414-015-1226-y 26162598

[125] Maroñas O, Phillips C, Söchtig J, et al. Development of a forensic skin colour predictive test. Forensic Sci Int Genet. 2014;13:34–44. 25082135 10.1016/j.fsigen.2014.06.017

[126] Xavier C, de la Puente M, Mosquera-Miguel A, et al. Development and validation of the VISAGE AmpliSeq basic tool to predict appearance and ancestry from DNA. Forensic Sci Int Genet. 2020;48:102336. 32619960 10.1016/j.fsigen.2020.102336

[127] Qiagen NV, Verogen Inc. ForenSeq DNA Signature Prep Kit. Last revision: 2022. Available from: https://verogen.com/products/forenseq-dna-signature-prep-kit/

[128] Keating B, Bansal AT, Walsh S, et al. First all-in-one diagnostic tool for DNA intelligence: genome-wide inference of biogeographic ancestry, appearance, relatedness, and sex with the Identitas v1 Forensic Chip. Int J Leg Med. 2013;127:559–572. 10.1007/s00414-012-0788-1 PMC3631519 23149900

[129] Identitas. IDentify. Last revision: 2022. Available from: https://www.identitascorp.com/identify-advantages/

[130] Parabon Nanolabs. DNA Phenotyping - Parabon® Snapshot® DNA Analysis Service. Last revision: 2024. Available from: https://snapshot.parabon-nanolabs.com/phenotyping

[131] Wienroth M . Governing anticipatory technology practices. Forensic DNA phenotyping and the forensic genetics community in Europe. New Genet Soc. 2018;37:137–152.

[132] Samuel G, Prainsack B. Forensic DNA phenotyping in Europe: views “on the ground” from those who have a professional stake in the technology. New Genetics and Society. 2018;38:119–141.

[133] Koops BJ, Schellekens MHM. Forensic DNA phenotyping: regulatory issues. Colum Sci & Tech. 2008;9:158–202.

[134] Granja R, Machado H. Forensic DNA phenotyping and its politics of legitimation and contestation: views of forensic geneticists in Europe. Soc Stud Sci. 2023;53:850–868. 10.1177/0306312720945033 PMC10696903 32729409

[135] Samuel G, Carmen Howard H, Cornel M, et al. A response to the forensic genetics policy initiative’s report ‘establishing best practice for forensic DNA databases’. Forensic Sci Int Genet. 2018;36:e19–e21. 30030012 10.1016/j.fsigen.2018.07.002

[136] Kidd JR, Friedlaender FR, Speed WC, et al. Analyses of a set of 128 ancestry informative single-nucleotide polymorphisms in a global set of 119 population samples. Investigative Genet. 2011;2:1. 10.1186/2041-2223-2-1 PMC3025953 21208434

[137] Scientific Working Group on DNA Analysis Methods (SWGDAM). Available from: https://www.swgdam.org/

[138] Coquet M, Terrado-Ortuño N. Forensic DNA phenotyping: privacy breach, bias reification, and the pitfalls of abstract assessments of rights. Int J Police Sci Manag. 2023;25:262–279.

[139] Katsara MA, Branicki W, Walsh S, et al. Evaluation of supervised machine-learning methods for predicting appearance traits from DNA. Forensic Sci Int Genet. 2021;53:102507. 33831816 10.1016/j.fsigen.2021.102507

[140] Mengel-From J, Børsting C, Sanchez JJ, et al. Human eye colour and *HERC2*, *OCA2* and *MATP*. Forensic Sci Int Genet. 2010;4:323–328. 20457063 10.1016/j.fsigen.2009.12.004

[141] Meyer OS, Børsting C, Andersen JD. Perception of blue and brown eye colours for forensic DNA phenotyping. Forensic Sci Int Genet Suppl Ser. 2019;7:476–477.

[142] Pośpiech E, Draus-Barini J, Kupiec T, et al. Prediction of eye color from genetic data using Bayesian approach. J Forensic Sci. 2012;57:880–886. 22372960 10.1111/j.1556-4029.2012.02077.x

[143] Dembinski GM, Picard CJ. Evaluation of the IrisPlex DNA-based eye color prediction assay in a United States population. Forensic Sci Int Genet. 2014;9:111–117. 24528589 10.1016/j.fsigen.2013.12.003

[144] Dario P, Mouriño H, Oliveira AR, et al. Assessment of IrisPlex-based multiplex for eye and skin color prediction with application to a Portuguese population. Int J Leg Med. 2015;129:1191–1200. 10.1007/s00414-015-1248-5 26289415

[145] Andersen JD, Johansen P, Harder S, et al. Genetic analyses of the human eye colours using a novel objective method for eye colour classification. Forensic Sci Int Genet. 2013;7:508–515. 23948321 10.1016/j.fsigen.2013.05.003

[146] Wollstein A, Walsh S, Liu F, et al. Novel quantitative pigmentation phenotyping enhances genetic association, epistasis, and prediction of human eye colour. Sci Rep. 2017;7:1–11. 28240252 10.1038/srep43359 PMC5327401

[147] Salas A, Phillips C, Carracedo A. Ancestry *vs* physical traits: the search for ancestry informative markers (AIMs). Int J Leg Med. 2005;120:188–189. 10.1007/s00414-005-0032-3 16133562

[148] Wendt FR, Churchill JD, Novroski NMM, et al. Genetic analysis of the Yavapai native Americans from West-Central Arizona using the Illumina MiSeq FGxTM forensic genomics system. Forensic Sci Int Genet. 2016;24:18–23. 27243782 10.1016/j.fsigen.2016.05.008

[149] Rajeevan H, Soundararajan U, Pakstis AJ, et al. Introducing the Forensic Research/Reference on Genetics knowledge base, FROG-kb. Investigative Genet. 2012;3:18. 10.1186/2041-2223-3-18 PMC3488007 22938150

[150] Kidd KK, Soundararajan U, Rajeevan H, et al. The redesigned Forensic Research/Reference on Genetics knowledge base, FROG-kb. Forensic Sci Int Genet. 2018;33:33–37. 29175726 10.1016/j.fsigen.2017.11.009

[151] Kersbergen P, van Duijn K, Kloosterman AD, et al. Developing a set of ancestry-sensitive DNA markers reflecting continental origins of humans. BMC Genet. 2009;10:69. 19860882 10.1186/1471-2156-10-69 PMC2775748

[152] Giardina E, Pietrangeli I, Martínez-Labarga C, et al. Haplotypes in *SLC24A5* gene as ancestry informative markers in different populations. Curr Genomics. 2008;9:110–114. 19440451 10.2174/138920208784139528 PMC2674805

[153] Pośpiech E, Wojas-Pelc A, Walsh S, et al. The common occurrence of epistasis in the determination of human pigmentation and its impact on DNA-based pigmentation phenotype prediction. Forensic Sci Int Genet. 2014;11:64–72. 24681889 10.1016/j.fsigen.2014.01.012

[154] Branicki W, Liu F, van Duijn K, et al. Model-based prediction of human hair color using DNA variants. Hum Genet. 2011;129:443–454. 21197618 10.1007/s00439-010-0939-8 PMC3057002

[155] Marcińska M, Pośpiech E, Abidi S, et al. Evaluation of DNA variants associated with androgenetic alopecia and their potential to predict male pattern baldness. PloS One. 2015;10:e0127852. 26001114 10.1371/journal.pone.0127852 PMC4441445

[156] Phillips C, Fondevila M, Lareau MV. A 34-plex autosomal SNP single base extension assay for ancestry investigations. Methods Mol Biol. 2012;830:109–126. 22139656 10.1007/978-1-61779-461-2_8

[157] de la Puente M, Santos C, Fondevila M, et al. The Global AIMs Nano set: a 31-plex SNaPshot assay of ancestry-informative SNPs. Forensic Sci Int Genet. 2016;22:81–88. 26881328 10.1016/j.fsigen.2016.01.015

[158] Bardan F, Higgins D, Austin JJ. A mini-multiplex SNaPshot assay for the triage of degraded human DNA. Forensic Sci Int Genet. 2018;34:62–70. 29428889 10.1016/j.fsigen.2018.02.006

[159] Muro T, Iida R, Fujihara J, et al. Simultaneous determination of seven informative Y chromosome SNPs to differentiate East Asian, European, and African populations. Leg Med. 2011;13:134–141. 10.1016/j.legalmed.2011.01.001 21315645

[160] Young JM, Martin B, Kanokwongnuwut P, et al. Detection of forensic identification and intelligence SNP data from latent DNA using three commercial MPS panels. Forensic Sci Int Genet Suppl Ser. 2019;7:864–865.

[161] Liu F, Hendriks AEJ, Ralf A, et al. Common DNA variants predict tall stature in Europeans. Hum Genet. 2014;133:587–597. 24253421 10.1007/s00439-013-1394-0

[162] Liu F, Zhong K, Jing X, et al. Update on the predictability of tall stature from DNA markers in Europeans. Forensic Sci Int Genet. 2019;42:8–13. 31207428 10.1016/j.fsigen.2019.05.006

[163] Rogalla U, Rychlicka E, Derenko MV, et al. Simple and cost-effective 14-loci SNP assay designed for differentiation of European, East Asian and African samples. Forensic Sci Int Genet. 2015;14:42–49. 25286442 10.1016/j.fsigen.2014.09.009

[164] Pośpiech E, Karłowska-Pik J, Marcińska M, et al. Evaluation of the predictive capacity of DNA variants associated with straight hair in Europeans. Forensic Sci Int Genet. 2015;19:280–288. 26414620 10.1016/j.fsigen.2015.09.004

[165] Liu F, Hamer MA, Heilmann S, et al. Prediction of male-pattern baldness from genotypes. Eur J Hum Genet. 2015;24:895–902. 26508577 10.1038/ejhg.2015.220 PMC4867459

[166] Qu Y, Tran D, Martinez-Marroquin E. Biogeographical Ancestry Inference from Genotype: A Comparison of Ancestral Informative SNPs and Genome-wide SNPs. In: Proceedings of the 2020 IEEE Symposium series on computational intelligence (SSCI); 2020 Dec 1–4; Canberra. 2020. p. 64–70.

[167] Jin XY, Wei YY, Lan Q, et al. A set of novel SNP loci for differentiating continental populations and three Chinese populations. PeerJ. 2019;7:e6508. 30956897 10.7717/peerj.6508 PMC6445247

[168] Truelsen DM, Farzad MS, Mogensen HS, et al. Typing of two Middle Eastern populations with the precision ID ancestry panel. Forensic Sci Int Genet Suppl Ser. 2017;6:e301–e302.

[169] Daniel R, Sanchez JJ, Nassif NT, et al. SNPs associated with physical traits: a valuable tool for the inference of biogeographical ancestry. Forensic Sci Int Genet Suppl Ser. 2008;1:538–540.

[170] Poetsch M, Blöhm R, Harder M, et al. Prediction of people’s origin from degraded DNA—presentation of SNP assays and calculation of probability. Int J Leg Med. 2013;127:347–357. 10.1007/s00414-012-0728-0 22918435

[171] Gu Y, Yun L, Zhang L, et al. The potential forensic utility of two single nucleotide polymorphisms in predicting biogeographical ancestry. Forensic Sci Int Genet Suppl Ser. 2011;3:e105–e106.

[172] Soejima M, Koda Y. Population differences of two coding SNPs in pigmentation-related genes *SLC24A5* and *SLC45A2*. Int J Leg Med. 2007;121:36–39. 10.1007/s00414-006-0112-z 16847698

[173] Wetton JH, Tsang KW, Khan H. Inferring the population of origin of DNA evidence within the UK by allele-specific hybridization of Y-SNPs. Forensic Sci Int. 2005;152:45–53. 15878814 10.1016/j.forsciint.2005.03.009

[174] Yuasa I, Umetsu K, Watanabe G, et al. MATP polymorphisms in Germans and Japanese: the L374F mutation as a population marker for Caucasoids. Int J Leg Med. 2004;118:364–366. 10.1007/s00414-004-0490-z 15455243

[175] Hunter P . Uncharted waters: next-generation sequencing and machine learning software allow forensic sicente to expand into phenotype prediction from DNA samples. EMBO Rep. 2018;19:e45810. 29437774 10.15252/embr.201845810 PMC5836095

[176] Caliebe A, Walsh S, Liu F, et al. Likelihood ratio and posterior odds in forensic genetics: two sides of the same coin. Forensic Sci Int Genet. 2017;28:203–210. 28314239 10.1016/j.fsigen.2017.03.004

[177] Albert FW, Kruglyak L. The role of regulatory variation in complex traits and disease. Nat Rev Genet. 2015;16:197–212. 25707927 10.1038/nrg3891

[178] Bradbury C, Köttgen A, Staubach F. Off-target phenotypes in forensic DNA phenotyping and biogeographic ancestry inference: a resource. Forensic Sci Int Genet. 2019;38:93–104. 30391626 10.1016/j.fsigen.2018.10.010

[179] Venables SJ, Mehta B, Daniel R, et al. Assessment of high resolution melting analysis as a potential SNP genotyping technique in forensic casework. Electrophoresis. 2014;35:3036–3043. 25142205 10.1002/elps.201400089

[180] Ragazzo M, Puleri G, Errichiello V, et al. Evaluation of OpenArrayTM as a genotyping method for Forensic DNA phenotyping and human identification. Genes (Basel). 2021;12:1–10. 10.3390/genes12020221 PMC7913479 33546406

[181] Mehta B, Daniel R, McNevin D. HRM and SNaPshot as alternative forensic SNP genotyping methods. Forensic Sci Med Pathol. 2017;13:293–301. 28523436 10.1007/s12024-017-9874-5

[182] Oh S, Kim J, Park S, et al. Prediction of Y haplogroup by polymerase chain reaction-reverse blot hybridization assay. Genes Genomics. 2019;41:297–304. 30456526 10.1007/s13258-018-0761-6

[183] Lundsberg B, Johansen P, Børsting C, et al. Development and optimisation of five multiplex assays with 115 of the AIM SNPs from the EUROFORGEN AIMs set on the Sequenom® MassARRAY® system. Forensic Sci Int Genet Suppl Ser. 2013;4:e182–e183.

[184] Hwa HL, Lin CP, Huang TY, et al. A panel of 130 autosomal single-nucleotide polymorphisms for ancestry assignment in five Asian populations and in Caucasians. Forensic Sci Med Pathol. 2017;13:177–187. 28439786 10.1007/s12024-017-9863-8

[185] Ribeiro J, Pereira V, Kondili A, et al. Typing of 111 ancestry informative markers in an Albanian population. Forensic Sci Int Genet Suppl Ser. 2015;5:e124–e125.

[186] Ren P, Liu J, Zhao H, et al. Construction of a rapid microfluidic-based SNP genotyping (MSG) chip for ancestry inference. Forensic Sci Int Genet. 2019;41:145–151. 31103798 10.1016/j.fsigen.2019.04.006

[187] Daniel R, Santos C, Phillips C, et al. A SNaPshot of next generation sequencing for forensic SNP analysis. Forensic Sci Int Genet. 2015;14:50–60. 25282603 10.1016/j.fsigen.2014.08.013

[188] Mehta B, Daniel R, Phillips C, et al. Massively parallel sequencing of customised forensically informative SNP panels on the MiSeq. Electrophoresis. 2016;37:2832–2840. 27605155 10.1002/elps.201600190

[189] Breslin K, Wills B, Ralf A, et al. HIrisPlex-S system for eye, hair, and skin color prediction from DNA: massively parallel sequencing solutions for two common forensically used platforms. Forensic Sci Int Genet. 2019;43:102152. 31518964 10.1016/j.fsigen.2019.102152

[190] Yang Y, Xie B, Yan J. Application of next-generation sequencing technology in forensic science. Genomics Proteomics Bioinformatics. 2014;12:190–197. 25462152 10.1016/j.gpb.2014.09.001 PMC4411420

[191] Ambers AD, Churchill JD, King JL, et al. More comprehensive forensic genetic marker analyses for accurate human remains identification using massively parallel DNA sequencing. BMC Genomics. 2016;17:21–30. 27766958 10.1186/s12864-016-3087-2 PMC5073988

[192] Jäger AC, Alvarez ML, Davis CP, et al. Developmental validation of the MiSeq FGx forensic genomics system for targeted next generation sequencing in forensic DNA casework and database laboratories. Forensic Sci Int Genet. 2017;28:52–70. 28171784 10.1016/j.fsigen.2017.01.011

[193] Turchi C, Onofri V, Melchionda F, et al. Development of a forensic DNA phenotyping panel using massive parallel sequencing. Forensic Sci Int Genet Suppl Ser. 2019;7:177–179.

[194] Melchionda F, Silvestrini B, Robino C, et al. Development and validation of MPS-based system for human appearance prediction in challenging forensic samples. Genes (Basel). 2022;13:1688. 10.3390/genes13101688 PMC9601425 36292573

[195] Young JM, Power D, Kanokwongnuwut P, et al. Ancestry and phenotype predictions from touch DNA using massively parallel sequencing. Int J Leg Med. 2021;135:81–89. 10.1007/s00414-020-02398-7 32815052

[196] Ralf A, Kayser M. Investigative DNA analysis of two-person mixed crime scene trace in a murder case. Forensic Sci Int Genet. 2021;54:102557. 34175530 10.1016/j.fsigen.2021.102557

[197] Diepenbroek M, Bayer B, Anslinger K. Pushing the boundaries: forensic DNA phenotyping challenged by single-cell sequencing. Genes (Basel). 2021;12:1362. 10.3390/genes12091362 PMC8466929 34573344

[198] Kukla-Bartoszek M, Teisseyre P, Pośpiech E, et al. Searching for improvements in predicting human eye colour from DNA. Int J Leg Med. 2021;135:2175–2187. 10.1007/s00414-021-02645-5 PMC8523394 34259936

[199] Cheung EYY, Gahan ME, McNevin D. Prediction of biogeographical ancestry in admixed individuals. Forensic Sci Int Genet. 2018;36:104–111. 29986229 10.1016/j.fsigen.2018.06.013

[200] Katsara MA, Branicki W, Pośpiech E, et al. Testing the impact of trait prevalence priors in Bayesian-based genetic prediction modelling of human appearance traits. Forensic Sci Int Genet. 2021;50:102412. 33260052 10.1016/j.fsigen.2020.102412

[201] Kastelic V, Drobnič K. A single-nucleotide polymorphism (SNP) multiplex system: the association of five SNPs with human eye and hair color in the Slovenian population and comparison using a Bayesian network and logistic regression model. Croat Med J. 2012;53:401–408. 23100201 10.3325/cmj.2012.53.401 PMC3490452

[202] Zaorska K, Zawierucha P, Nowicki M. Prediction of skin color, tanning and freckling from DNA in Polish population: linear regression, random forest and neural network approaches. Hum Genet. 2019;138:635–647. 30980179 10.1007/s00439-019-02012-w PMC6554257

[203] Zidkova A, Horinek A, Stenzl V, et al. Application of multifactor dimensionality reduction analysis and Bayesian networks for eye color and ancestry prediction for forensic purposes in the Czech Republic. Forensic Sci Int Genet Suppl Ser. 2013;4:e322–e323.

[204] Pfaffelhuber P, Rohde A. A central limit theorem concerning uncertainty in estimates of individual admixture. Theor Popul Biol. 2022;148:28–39. 36208800 10.1016/j.tpb.2022.09.003

[205] Bulbul O, Filoglu G, Zorlu T, et al. Inference of biogeographical ancestry across central regions of Eurasia. Int J Leg Med. 2016;130:73–79. 10.1007/s00414-015-1246-7 26289413

[206] Pośpiech E, Draus-Barini J, Kupiec T, et al. Gene-gene interactions contribute to eye colour variation in humans. J Hum Genet. 2011;56:447–455. 21471978 10.1038/jhg.2011.38

[207] Palmal S, Adhikari K, Mendoza-Revilla J, et al. Prediction of eye, hair and skin colour in Latin Americans. Forensic Sci Int Genet. 2021;53:102517. 33865096 10.1016/j.fsigen.2021.102517

[208] Salvoro C, Faccinetto C, Zucchelli L, et al. Performance of four models for eye color prediction in an Italian population sample. Forensic Sci Int Genet. 2019;40:192–200. 30884346 10.1016/j.fsigen.2019.03.008

[209] Meyer OS, Salvo NM, Kjærbye A, et al. Prediction of eye colour in Scandinavians using the EyeColour 11 (EC11) SNP set. Genes (Basel). 2021;12:821. 10.3390/genes12060821 PMC8227851 34071952

[210] Caliebe A, Harder M, Schuett R, et al. The more the merrier? How a few SNPs predict pigmentation phenotypes in the Northern German population. Eur J Hum Genet. 2015;24:739–747. 26286644 10.1038/ejhg.2015.167 PMC4930077

[211] Pfaffelhuber P, Sester-Huss E, Baumdicker F, et al. Inference of recent admixture using genotype data. Forensic Sci Int Genet. 2022;56:102593. 34735936 10.1016/j.fsigen.2021.102593

[212] Walsh S, Wollstein A, Liu F, et al. DNA-based eye colour prediction across Europe with the IrisPlex system. Forensic Sci Int Genet. 2012;6:330–340. 21813346 10.1016/j.fsigen.2011.07.009

[213] Caliebe A, Krawczak M, Kayser M. Predictive values in forensic DNA phenotyping are not necessarily prevalence-dependent. Forensic Sci Int Genet. 2018;33:e7–e8. 29157933 10.1016/j.fsigen.2017.11.006

[214] Pereira V, Mogensen HS, Børsting C, et al. Evaluation of the Precision ID ancestry panel for crime case work: a SNP typing assay developed for typing of 165 ancestral informative markers. Forensic Sci Int Genet. 2017;28:138–145. 28273506 10.1016/j.fsigen.2017.02.013

[215] Hussing C, Huber C, Bytyci R, et al. Sequencing of 231 forensic genetic markers using the MiSeq FGxTM forensic genomics system—an evaluation of the assay and software. Forensic Sci Res. 2018;3:111–123. 30483659 10.1080/20961790.2018.1446672 PMC6197110

[216] Brión M, Sanchez JJ, Balogh K, et al. Analysis of 29 Y-chromosome SNPs in a single multiplex useful to predict the geographic origin of male lineages. Int Congr Ser. 2006;1288:13–15.

[217] Brión M, Sanchez JJ, Balogh K, et al. Introduction of a single nucleotide polymorphism-based ‘major Y-chromosome haplogroup typing kit’ suitable for predicting the geographical origin of male lineages. Electrophoresis. 2005;26:4411–4420. 16273584 10.1002/elps.200500293

[218] Lessig R, Edelmann J, Thiele K, et al. Results of Y-SNP typing in three different populations. Forensic Sci Int Genet Suppl Ser. 2008;1:219–221.

[219] Brión M, Sobrino B, Blanco-Verea A, et al. Hierarchical analysis of 30 Y-chromosome SNPs in European populations. Int J Leg Med. 2005;119:10–15. 10.1007/s00414-004-0439-2 15095093

[220] Onofri V, Alessandrini F, Turchi C, et al. Development of multiplex PCRs for evolutionary and forensic applications of 37 human Y chromosome SNPs. Forensic Sci Int. 2006;157:23–35. 15896936 10.1016/j.forsciint.2005.03.014

[221] Bouakaze C, Keyser C, Amory S, et al. First successful assay of Y-SNP typing by SNaPshot minisequencing on ancient DNA. Int J Leg Med. 2007;121:493–499. 10.1007/s00414-007-0177-3 17534642

[222] Chiurillo MA, Lander N, Rojas M, et al. Development of Y-SNP typing assay for forensic application in Venezuelan population. Forensic Sci Int Genet Suppl Ser. 2009;2:444–445.

[223] Noveski P, Trivodalieva S, Efremov GD, et al. Y chromosome single nucleotide polymorphisms typing by SNaPshot MINISEQUENCING. Balk J Med Genet. 2010;13:9–16.

[224] van Oven M, Ralf A, Kayser M. An efficient multiplex genotyping approach for detecting the major worldwide human Y-chromosome haplogroups. Int J Leg Med. 2011;125:879–885. 10.1007/s00414-011-0605-2 PMC3192277 21785904

[225] Ralf A, van Oven M, Montiel González D, et al. Forensic Y-SNP analysis beyond SNaPshot: high-resolution Y-chromosomal haplogrouping from low quality and quantity DNA using ion AmpliSeq and targeted massively parallel sequencing. Forensic Sci Int Genet. 2019;41:93–106. 31063905 10.1016/j.fsigen.2019.04.001

[226] McNevin D, Bate A, Daniel R, et al. A preliminary mitochondrial DNA SNP genotyping assay for inferring genealogy. Aust J Forensic Sci. 2011;43:39–51.

[227] van Oven M, Vermeulen M, Kayser M. Multiplex genotyping system for efficient inference of matrilineal genetic ancestry with continental resolution. Investigative Genet. 2011;2:6. 10.1186/2041-2223-2-6 PMC3078086 21429198

[228] Ballantyne KN, van Oven M, Ralf A, et al. MtDNA SNP multiplexes for efficient inference of matrilineal genetic ancestry within Oceania. Forensic Sci Int Genet. 2012;6:425–436. 21940232 10.1016/j.fsigen.2011.08.010

[229] Chaitanya L, van Oven M, Weiler N, et al. Developmental validation of mitochondrial DNA genotyping assays for adept matrilineal inference of biogeographic ancestry at a continental level. Forensic Sci Int Genet. 2014;11:39–51. 24631695 10.1016/j.fsigen.2014.02.010

[230] Palencia-Madrid L, Vinueza-Espinosa D, Baeta M, et al. Validation of a 52-mtSNP minisequencing panel for haplogroup classification of forensic DNA samples. Int J Leg Med. 2020;134:929–936. 10.1007/s00414-020-02264-6 32030455

[231] Daniel R, Walsh SJ, Piper A. Investigation of single-nucleotide polymorphisms associated with ethnicity. Int Congr Ser. 2006;1288:79–81.

[232] Fondevila M, Phillips C, Santos C, et al. Revision of the SNPforID 34-plex forensic ancestry test: assay enhancements, standard reference sample genotypes and extended population studies. Forensic Sci Int Genet. 2013;7:63–74. 22749789 10.1016/j.fsigen.2012.06.007

[233] Phillips C, Fondevila M, Vallone PM, et al. Characterization of U.S. population samples using a 34plex ancestry informative SNP multiplex. Forensic Sci Int Genet Suppl Ser. 2011;3:e182–e183.

[234] Khodjet-El-Khil H, Fadhlaoui-Zid K, Cherni L, et al. Genetic analysis of the SNPforID 34-plex ancestry informative SNP panel in Tunisian and Libyan populations. Forensic Sci Int Genet. 2011;5:e45–e47. 20850402 10.1016/j.fsigen.2010.07.007

[235] Prestes PR, Mitchell RJ, Santos C, et al. The SNPforID 34-plex—its ability to infer level of admixture in individuals. Forensic Sci Int Genet Suppl Ser. 2013;4:e13–e14.

[236] Santos C, Fondevila M, Ballard D, et al. Forensic ancestry analysis with two capillary electrophoresis ancestry informative marker (AIM) panels: results of a collaborative EDNAP exercise. Forensic Sci Int Genet. 2015;19:56–67. 26122263 10.1016/j.fsigen.2015.06.004

[237] Gomes C, Fondevila M, Palomo-Díez S, et al. Phenotyping the ancient world: the physical appearance and ancestry of very degraded samples from a chalcolithic human remains. Forensic Sci Int Genet Suppl Ser. 2017;6:e484–e486.

[238] Daniel R, Sanchez JJ, Nassif NT, et al. Partial forensic validation of a 16plex SNP assay for the inference of biogeographical ancestry. Forensic Sci Int Genet Suppl Ser. 2009;2:477–478.

[239] Eduardoff M, Gross TE, Santos C, et al. Interlaboratory evaluation of the EUROFORGEN Global ancestry-informative SNP panel by massively parallel sequencing using the ion PGM™. Forensic Sci Int Genet. 2016;23:178–189. 27208666 10.1016/j.fsigen.2016.04.008

[240] Bulbul O, Filoglu G. Development of a SNP panel for predicting biogeographical ancestry and phenotype using massively parallel sequencing. Electrophoresis. 2018;39:2743–2751. 30091798 10.1002/elps.201800243

[241] Daca-Roszak P, Pfeifer A, Zebracka-Gala J, et al. EurEAs_Gplex—a new SNaPshot assay for continental population discrimination and gender identification. Forensic Sci Int Genet. 2016;20:89–100. 26520215 10.1016/j.fsigen.2015.10.004

[242] Bulbul O, Cherni L, Khodjet-El-Khil H, et al. Evaluating a subset of ancestry informative SNPs for discriminating among Southwest Asian and circum-Mediterranean populations. Forensic Sci Int Genet. 2016;23:153–158. 27160361 10.1016/j.fsigen.2016.04.010

[243] Li CX, Pakstis AJ, Jiang L, et al. A panel of 74 AISNPs: improved ancestry inference within Eastern Asia. Forensic Sci Int Genet. 2016;23:101–110. 27077960 10.1016/j.fsigen.2016.04.002

[244] Yuasa I, Akane A, Yamamoto T, et al. Japaneseplex: a forensic SNP assay for identification of Japanese people using Japanese-specific alleles. Leg Med. 2018;33:17–22. 10.1016/j.legalmed.2018.04.008 29705644

[245] Freire-Aradas A, Ruiz Y, Phillips C, et al. Exploring iris colour prediction and ancestry inference in admixed populations of South America. Forensic Sci Int Genet. 2014;13:3–9. 25051225 10.1016/j.fsigen.2014.06.007

[246] Gross TE, Zaumsegel D, Rothschild MA, et al. Combined analysis of two different ancestry informative assays using SNPs and Indels in Eurasian populations. Forensic Sci Int: Genet Suppl. 2013;4:e25–e26.

[247] Phillips C, McNevin D, Kidd KK, et al. MAPlex—a massively parallel sequencing ancestry analysis multiplex for Asia-Pacific populations. Forensic Sci Int Genet. 2019;42:213–226. 31377479 10.1016/j.fsigen.2019.06.022

[248] Cheung EYY, Phillips C, Eduardoff M, et al. Performance of ancestry-informative SNP and microhaplotype markers. Forensic Sci Int Genet. 2019;43:102141. 31442930 10.1016/j.fsigen.2019.102141

[249] Xavier C, de la Puente M, Phillips C, et al. Forensic evaluation of the Asia Pacific ancestry-informative MAPlex assay. Forensic Sci Int Genet. 2020;48:102344. 32615397 10.1016/j.fsigen.2020.102344

[250] Espregueira Themudo G, Smidt Mogensen H, Børsting C, et al. Frequencies of HID-ion AmpliSeq ancestry panel markers among Greenlanders. Forensic Sci Int Genet. 2016;24:60–64. 27326551 10.1016/j.fsigen.2016.06.001

[251] García O, Ajuriagerra JA, Alday A, et al. Frequencies of the precision ID ancestry panel markers in Basques using the ion torrent PGM™ platform. Forensic Sci Int Genet. 2017;31:e1–e4. 28935228 10.1016/j.fsigen.2017.09.006

[252] Nakanishi H, Pereira V, Børsting C, et al. Analysis of mainland Japanese and Okinawan Japanese populations using the precision ID ancestry panel. Forensic Sci Int Genet. 2018;33:106–109. 29223883 10.1016/j.fsigen.2017.12.004

[253] Wang Z, He G, Luo T, et al. Massively parallel sequencing of 165 ancestry informative SNPs in two Chinese Tibetan-Burmese minority ethnicities. Forensic Sci Int Genet. 2018;34:141–147. 29477877 10.1016/j.fsigen.2018.02.009

[254] Pereira V, Santangelo R, Børsting C, et al. Evaluation of the Precision of ancestry inferences in South American admixed populations. Front Genet. 2020;11:966. 32973885 10.3389/fgene.2020.00966 PMC7472784

[255] Xie T, Shen C, Liu C, et al. Ancestry inference and admixture component estimations of Chinese Kazak group based on 165 AIM-SNPs *via* NGS platform. J Hum Genet. 2020;65:461–468. 32081902 10.1038/s10038-020-0725-y

[256] He G, Liu J, Wang M, et al. Massively parallel sequencing of 165 ancestry-informative SNPs and forensic biogeographical ancestry inference in three Southern Chinese Sinitic/Tai-Kadai populations. Forensic Sci Int Genet. 2021;52:102475. 33561661 10.1016/j.fsigen.2021.102475

[257] Cooley AM, Meiklejohn KA, Damaso N, et al. Performance comparison of massively parallel sequencing (MPS) instruments using single-nucleotide polymorphism (SNP) panels for ancestry. SLAS Technol. 2021;26:103–112. 32914686 10.1177/2472630320954180

[258] Shan MA, Meyer OS, Refn M, et al. Analysis of skin pigmentation and genetic ancestry in three subpopulations from Pakistan: Punjabi, Pashtun, and Baloch. Genes (Basel). 2021;12:733. 34068188 10.3390/genes12050733 PMC8152963

[259] Jin S, Chase M, Henry M, et al. Implementing a biogeographic ancestry inference service for forensic casework. Electrophoresis. 2018;39:2757–2765. 30125362 10.1002/elps.201800171

[260] Mogensen HS, Tvedebrink T, Børsting C, et al. Ancestry prediction efficiency of the software GenoGeographer using a z-score method and the ancestry informative markers in the Precision ID ancestry panel. Forensic Sci Int Genet. 2020;44:102154. 31670023 10.1016/j.fsigen.2019.102154

[261] Walsh S, Lindenbergh A, Zuniga SB, et al. Developmental validation of the IrisPlex system: determination of blue and brown iris colour for forensic intelligence. Forensic Sci Int Genet. 2011;5:464–471. 20947461 10.1016/j.fsigen.2010.09.008

[262] Chaitanya L, Walsh S, Andersen JD, et al. Collaborative EDNAP exercise on the IrisPlex system for DNA-based prediction of human eye colour. Forensic Sci Int Genet. 2014;11:241–251. 24880832 10.1016/j.fsigen.2014.04.006

[263] Prestes PR, Mitchell RJ, Daniel R, et al. Evaluation of the IrisPlex system in admixed individuals. Forensic Sci Int Genet Suppl Ser. 2011;3:e283–e284.

[264] Purps J, Geppert M, Nagy M, et al. Evaluation of the IrisPlex eye colour prediction tool in a German population sample. Forensic Sci Int Genet Suppl Ser. 2011;3:e202–e203.

[265] Pneuman A, Budimlija ZM, Caragine T, et al. Verification of eye and skin color predictors in various populations. Leg Med. 2012;14:78–83. 10.1016/j.legalmed.2011.12.005 22284939

[266] Martinez-Cadenas C, Peña-Chilet M, Llorca-Cardeñosa MJ, et al. Gender and eye colour prediction discrepancies: a reply to criticisms. Forensic Sci Int Genet. 2014;9:e7–e9. 24216441 10.1016/j.fsigen.2013.10.002

[267] Liu F, Walsh S, Kayser M. Of sex and IrisPlex eye colour prediction: a reply to Martinez-Cadenas et al. Forensic Sci Int Genet. 2014;9:e5–e6. 23845206 10.1016/j.fsigen.2013.06.006

[268] Pośpiech E, Karłowska-Pik J, Ziemkiewicz B, et al. Further evidence for population specific differences in the effect of DNA markers and gender on eye colour prediction in forensics. Int J Leg Med. 2016;130:923–934. 10.1007/s00414-016-1388-2 PMC4912978 27221533

[269] Yun L, Gu Y, Rajeevan H, et al. Application of six IrisPlex SNPs and comparison of two eye color prediction systems in diverse Eurasia populations. Int J Leg Med. 2014;128:447–453. 10.1007/s00414-013-0953-1 24395150

[270] Bulbul O, Zorlu T, Filoglu G. Prediction of human eye colour using highly informative phenotype SNPs (PISNPs). Aus J Forensic Sci. 2018;52:27–37.

[271] Al-Rashedi NAM, Mandal AM, ALObaidi LA. Eye color prediction using the IrisPlex system: a limited pilot study in the Iraqi population. Egypt J Forensic Sci. 2020;10:1–6.

[272] Liu F, van Duijn K, Vingerling JR, et al. Eye color and the prediction of complex phenotypes from genotypes. Curr Biol. 2009;19:R192–R193. 19278628 10.1016/j.cub.2009.01.027

[273] Kastelic V, Pośpiech E, Draus-Barini J, et al. Prediction of eye color in the Slovenian population using the IrisPlex SNPs. Croat Med J. 2013;54:381–386. 23986280 10.3325/cmj.2013.54.381 PMC3760663

[274] Paparazzo E, Gozalishvili A, Lagani V, et al. A new approach to broaden the range of eye colour identifiable by IrisPlex in DNA phenotyping. Sci Rep. 2022;12:1–10. 35896692 10.1038/s41598-022-17208-w PMC9329466

[275] Allwood JS, Harbison SA. SNP model development for the prediction of eye colour in New Zealand. Forensic Sci Int Genet. 2013;7:444–452. 23597786 10.1016/j.fsigen.2013.03.005

[276] Shapturenko MN, Vakula SI, Kandratsiuk AV, et al. *HERC2* (rs12913832) and *OCA2* (rs1800407) genes polymorphisms in relation to iris color variation in Belarusian population. Forensic Sci Int Genet Suppl Ser. 2019;7:331–332.

[277] Alghamdi J, Amoudi M, Kassab AC, et al. Eye color prediction using single nucleotide polymorphisms in Saudi population. Saudi J Biol Sci. 2019;26:1607–1612. 31762634 10.1016/j.sjbs.2018.09.011 PMC6864217

[278] Walsh S, Kayser M. A practical guide to the HIrisPlex system: simultaneous prediction of eye and hair color from DNA. Methods Mol Biol. 2016;1420:213–231. 27259743 10.1007/978-1-4939-3597-0_17

[279] Draus-Barini J, Walsh S, Pośpiech E, et al. Bona fide colour: DNA prediction of human eye and hair colour from ancient and contemporary skeletal remains. Investigative Genet. 2013;4:1–15. 10.1186/2041-2223-4-3 PMC3551694 23317428

[280] Walsh S, Chaitanya L, Clarisse L, et al. Developmental validation of the HIrisPlex system: DNA-based eye and hair colour prediction for forensic and anthropological usage. Forensic Sci Int Genet. 2014;9:150–161. 24528593 10.1016/j.fsigen.2013.12.006

[281] Chaitanya L, Pajnič IZ, Walsh S, et al. Bringing colour back after 70 years: predicting eye and hair colour from skeletal remains of World War II victims using the HIrisPlex system. Forensic Sci Int Genet. 2017;26:48–57. 27780108 10.1016/j.fsigen.2016.10.004

[282] Zupanič PI . Identification of a Slovenian prewar elite couple killed in the Second World War. Forensic Sci Int. 2021;327:110994. 34536754 10.1016/j.forsciint.2021.110994

[283] Kukla-Bartoszek M, Pośpiech E, Spólnicka M, et al. Investigating the impact of age-depended hair colour darkening during childhood on DNA-based hair colour prediction with the HIrisPlex system. Forensic Sci Int Genet. 2018;36:26–33. 29913343 10.1016/j.fsigen.2018.06.007

[284] Carratto TMT, Marcorin L, do Valle-Silva G, et al. Prediction of eye and hair pigmentation phenotypes using the HIrisPlex system in a Brazilian admixed population sample. Int J Leg Med. 2021;135:1329–1339. 10.1007/s00414-021-02554-7 33884487

[285] Grimes EA, Noake PJ, Dixon L, et al. Sequence polymorphism in the human melanocortin 1 receptor gene as an indicator of the red hair phenotype. Forensic Sci Int. 2001;122:124–129. 11672965 10.1016/s0379-0738(01)00480-7

[286] Branicki W, Kupiec T, Wolańska-Nowak P, et al. Determination of forensically relevant SNPs in the *MC1R* gene. Int Congr Ser. 2006;1288:816–818.

[287] Branicki W, Brudnik U, Kupiec T, et al. Determination of phenotype associated SNPs in the *MC1R* gene. J Forensic Sci. 2007;52:349–354. 17316231 10.1111/j.1556-4029.2006.00361.x

[288] Branicki W, Wolańska-Nowak P, Brudnik U, et al. Forensic application of a rapid test for red hair colour prediction and sex determination. Z Zagadnien Nauk Sadowych. 2007;69:37–51.

[289] Marano LA, Andersen JD, Goncalves FT, et al. Evaluation of HIrisplex-S system markers for eye, skin and hair color prediction in an admixed Brazilian population. Forensic Sci Int Genet Suppl Ser. 2019;7:427–428.

[290] Carratto TMT, Marcorin L, Debortoli G, et al. Evaluation of the HIrisPlex-S system in a Brazilian population sample. Forensic Sci Int Genet Suppl Ser. 2019;7:794–796.

[291] Kukla-Bartoszek M, Szargut M, Pośpiech E, et al. The challenge of predicting human pigmentation traits in degraded bone samples with the MPS-based HIrisPlex-S system. Forensic Sci Int Genet. 2020;47:102301. 32387914 10.1016/j.fsigen.2020.102301

[292] Chen Y, Branicki W, Walsh S, et al. The impact of correlations between pigmentation phenotypes and underlying genotypes on genetic prediction of pigmentation traits. Forensic Sci Int Genet. 2021;50:102395. 33070049 10.1016/j.fsigen.2020.102395

[293] Gentile F, Cherubini A, Colloca D, et al. Evaluation of PyroMark Q48 autoprep with HIrisPlex-S in an Italian population sample. Forensic Sci Int Genet Suppl Ser. 2022;8:308–310.

[294] Valenzuela RK, Henderson MS, Walsh MH, et al. Predicting phenotype from genotype: normal pigmentation. J Forensic Sci. 2010;55:315–322. 20158590 10.1111/j.1556-4029.2009.01317.x PMC3626268

[295] Spichenok O, Budimlija ZM, Mitchell AA, et al. Prediction of eye and skin color in diverse populations using seven SNPs. Forensic Sci Int Genet. 2011;5:472–478. 21050833 10.1016/j.fsigen.2010.10.005

[296] Hart KL, Kimura SL, Mushailov V, et al. Improved eye- and skin-color prediction based on 8 SNPs. Croat Med J. 2013;54:248–256. 23771755 10.3325/cmj.2013.54.248 PMC3694299

[297] Mushailov V, Rodriguez SA, Budimlija ZM, et al. Assay development and validation of an 8-SNP multiplex test to predict eye and skin coloration. J Forensic Sci. 2015;60:990–1000. 25782558 10.1111/1556-4029.12758

[298] Lim S, Youn JP, Hong S, et al. Customized multiplexing SNP panel for Korean-specific DNA phenotyping in forensic applications. Genes Genomics. 2017;39:723–732.

[299] Liu F, Chen Y, Zhu G, et al. Meta-analysis of genome-wide association studies identifies 8 novel loci involved in shape variation of human head hair. Hum Mol Genet. 2018;27:559–575. 29220522 10.1093/hmg/ddx416 PMC5886212

[300] Pośpiech E, Karłowska-Pik J, Kukla-Bartoszek M, et al. Overlapping association signals in the genetics of hair-related phenotypes in humans and their relevance to predictive DNA analysis. Forensic Sci Int Genet. 2022;59:102693. 35398773 10.1016/j.fsigen.2022.102693

[301] Fagertun J, Wolffhechel K, Pers TH, et al. Predicting facial characteristics from complex polygenic variations. Forensic Sci Int Genet. 2015;19:263–268. 26355663 10.1016/j.fsigen.2015.08.004

[302] Qiao L, Yang Y, Fu P, et al. Genome-wide variants of Eurasian facial shape differentiation and a prospective model of DNA based face prediction. J Genet Genomics. 2018;45:419–432. 30174134 10.1016/j.jgg.2018.07.009

[303] Noreen S, Ballard D, Mehmood T, et al. Evaluation of loci to predict ear morphology using two SNaPshot assays. Forensic Sci Med Pathol. 2022;19:335–356. 36401782 10.1007/s12024-022-00545-7 PMC10518297

[304] Hussing C, Børsting C, Mogensen HS, et al. Testing of the Illumina® ForenSeq™ kit. Forensic Sci Int Genet Suppl Ser. 2015;5:e449–e450.

[305] Churchill JD, Schmedes SE, King JL, et al. Evaluation of the Illumina® Beta version ForenSeq™ DNA signature prep kit for use in genetic profiling. Forensic Sci Int Genet. 2016;20:20–29. 26433485 10.1016/j.fsigen.2015.09.009

[306] Churchill JD, Novroski NMM, King JL, et al. Population and performance analyses of four major populations with Illumina’s FGx forensic genomics system. Forensic Sci Int Genet. 2017;30:81–92. 28651097 10.1016/j.fsigen.2017.06.004

[307] Silvia AL, Shugarts N, Smith J. A preliminary assessment of the ForenSeq™ FGx system: next generation sequencing of an STR and SNP multiplex. Int J Leg Med. 2017;131:73–86. 10.1007/s00414-016-1457-6 27785563

[308] Sidstedt M, Junker K, Forsberg C, et al. In-house validation of MPS-based methods in a forensic laboratory. Forensic Sci Int Genet Suppl Ser. 2019;7:635–636.

[309] Sharma V, Jani K, Khosla P, et al. Evaluation of ForenSeq™ signature prep kit B on predicting eye and hair coloration as well as biogeographical ancestry by using Universal Analysis Software (UAS) and available web-tools. Electrophoresis. 2019;40:1353–1364. 30767247 10.1002/elps.201800344

[310] Frégeau CJ . Validation of the Verogen ForenSeq™ DNA signature prep kit/primer mix B for phenotypic and biogeographical ancestry predictions using the micro MiSeq® flow cells. Forensic Sci Int Genet. 2021;53:102533. 34058534 10.1016/j.fsigen.2021.102533

[311] Salvo NM, Janssen K, Kirsebom MK, et al. Predicting eye and hair colour in a Norwegian population using Verogen’s ForenSeq™ DNA signature prep kit. Forensic Sci Int Genet. 2022;56:102620. 34735941 10.1016/j.fsigen.2021.102620

[312] Weisz NA, Roberts KA, Hardy WR. Reliability of phenotype estimation and extended classification of ancestry using decedent samples. Int J Leg Med. 2021;135:2221–2233. 10.1007/s00414-021-02631-x 34436656

[313] Barbarić L, Horjan-Zanki I. Challenges in the recovery of the genetic data from human remains found on the Western Balkan migration route. Int J Leg Med. 2022;137:181–193. 10.1007/s00414-022-02829-7 35449468

[314] Frégeau CJ . A multiple predictive tool approach for phenotypic and biogeographical ancestry inferences. Can Soc Forensic Sci J. 2021;55:71–99.

[315] Junker K, Staadig A, Sidstedt M, et al. Phenotype prediction accuracy—a Swedish perspective. Forensic Sci Int Genet Suppl Ser. 2019;7:384–386.

[316] Magdalena M, Wróbel M, Parys-Proszek A, et al. Evaluation of the performance of the beta version of the ForenSeq DNA signature prep kit on the MiSeq FGx forensic genomics system. Forensic Sci Int Genet Suppl Ser. 2019;7:585–586.

[317] Guo F, Yu J, Zhang L, et al. Massively parallel sequencing of forensic STRs and SNPs using the Illumina® ForenSeq™ DNA signature prep kit on the MiSeq FGx™ forensic genomics system. Forensic Sci Int Genet. 2017;31:135–148. 28938154 10.1016/j.fsigen.2017.09.003

[318] Ramani A, Wong Y, Tan SZ, et al. Ancestry prediction in Singapore population samples using the Illumina ForenSeq kit. Forensic Sci Int Genet. 2017;31:171–179. 29040920 10.1016/j.fsigen.2017.08.013

[319] Bouakaze C, Keyser C, Crubézy E, et al. Pigment phenotype and biogeographical ancestry from ancient skeletal remains: inferences from multiplexed autosomal SNP analysis. Int J Leg Med. 2009;123:315–325. 10.1007/s00414-009-0348-5 19415315

[320] Butler K, Peck M, Hart J, et al. Molecular “eyewitness”: forensic prediction of phenotype and ancestry. Forensic Sci Int Genet Suppl Ser. 2011;3:e498–e499.

[321] Castel C, Piper A. Development of a SNP multiplex assay for the inference of biogeographical ancestry and pigmentation phenotype. Forensic Sci Int Genet Suppl Ser. 2011;3:e411–e412.

[322] Gettings KB, Lai R, Johnson JL, et al. A 50-SNP assay for biogeographic ancestry and phenotype prediction in the U.S. population. Forensic Sci Int Genet. 2014;8:101–108. 24315596 10.1016/j.fsigen.2013.07.010

[323] Bulbul O, Filoglu G, Altuncul H, et al. A SNP multiplex for the simultaneous prediction of biogeographic ancestry and pigmentation type. Forensic Sci Int Genet Suppl Ser. 2011;3:e500–e501.

[324] Palencia-Madrid L, Xavier C, de la Puente M, et al. Evaluation of the VISAGE basic tool for appearance and ancestry prediction using PowerSeq chemistry on the MiSeq FGx system. Genes. 2020;11:708. 32604780 10.3390/genes11060708 PMC7349024

[325] de la Puente M, Ruiz-Ramírez J, Ambroa-Conde A, et al. Development and evaluation of the ancestry informative marker panel of the VISAGE basic tool. Genes. 2021;12:1284. 34440458 10.3390/genes12081284 PMC8391248

[326] Xavier C, de la Puente M, Sidstedt M, et al. Evaluation of the VISAGE basic tool for appearance and ancestry inference using ForenSeq® chemistry on the MiSeq FGx® system. Forensic Sci Int Genet. 2022;58:102675. 35144074 10.1016/j.fsigen.2022.102675

[327] Diepenbroek M, Bayer B, Schwender K, et al. Evaluation of the ion AmpliSeq™ PhenoTrivium panel: MPS-based assay for ancestry and phenotype predictions challenged by casework samples. Genes. 2020;11:1398. 33255693 10.3390/genes11121398 PMC7760956

[328] Rauf S, Austin JJ, Higgins D, et al. Unveiling forensically relevant biogeographic, phenotype and Y-chromosome SNP variation in Pakistani ethnic groups using a customized hybridisation enrichment forensic intelligence panel. PloS One. 2022;17:e0264125. 35176104 10.1371/journal.pone.0264125 PMC8853543

[329] Fesenko DO, Ivanovsky ID, Ivanov PL, et al. A biochip for genotyping polymorphisms associated with eye, hair, skin color, ABO blood group, sex, Y chromosome core haplogroup, and its application to study the Slavic population. Mol Biol. 2022;56:780–799. 10.31857/S0026898422050056 36165022

